# Exploring the evolutionary origin of floral organs of *Erycina pusilla*, an emerging orchid model system

**DOI:** 10.1186/s12862-017-0938-7

**Published:** 2017-03-23

**Authors:** Anita Dirks-Mulder, Roland Butôt, Peter van Schaik, Jan Willem P. M. Wijnands, Roel van den Berg, Louie Krol, Sadhana Doebar, Kelly van Kooperen, Hugo de Boer, Elena M. Kramer, Erik F. Smets, Rutger A. Vos, Alexander Vrijdaghs, Barbara Gravendeel

**Affiliations:** 1 Endless Forms group, Naturalis Biodiversity Center, Vondellaan 55, 2332 AA Leiden, The Netherlands; 2 Faculty of Science and Technology, University of Applied Sciences Leiden, Zernikedreef 11, 2333 CK Leiden, The Netherlands; 3 Department of Organismic and Evolutionary Biology, Harvard University, 16 Divinity Ave, Cambridge, MA 02138 USA; 4 Institute for Biodiversity and Ecosystem Dynamics, University of Amsterdam, Science Park 904, 1098 XH Amsterdam, The Netherlands; 5 Institute Biology Leiden, Leiden University, Sylviusweg 72, 2333 BE Leiden, The Netherlands; 6 Ecology, Evolution and Biodiversity Conservation cluster, KU Leuven, Kasteelpark Arenberg 31, 3001 Leuven, Belgium; 7 The Natural History Museum, University of Oslo, P.O. Box 1172, Blindern, 0318 Oslo, Norway; 8 Department of Organismal Biology, Evolutionary Biology Centre, Uppsala University, Norbyvägen 18D, Uppsala, SE-75236 Sweden

**Keywords:** Deceptive pollination, Floral development, MADS-box genes, Mimicry, Vascular bundles

## Abstract

**Background:**

Thousands of flowering plant species attract pollinators without offering rewards, but the evolution of this deceit is poorly understood. Rewardless flowers of the orchid *Erycina pusilla* have an enlarged median sepal and incised median petal (‘lip’) to attract oil-collecting bees. These bees also forage on similar looking but rewarding Malpighiaceae flowers that have five unequally sized petals and gland-carrying sepals. The lip of *E. pusilla* has a ‘callus’ that, together with winged ‘stelidia’, mimics these glands. Different hypotheses exist about the evolutionary origin of the median sepal, callus and stelidia of orchid flowers.

**Results:**

The evolutionary origin of these organs was investigated using a combination of morphological, molecular and phylogenetic techniques to a developmental series of floral buds of *E. pusilla*. The vascular bundle of the median sepal indicates it is a first whorl organ but its convex epidermal cells reflect convergence of petaloid features. Expression of *AGL6 EpMADS4* and *APETALA3 EpMADS14* is low in the median sepal, possibly correlating with its petaloid appearance. A vascular bundle indicating second whorl derivation leads to the lip. *AGL6 EpMADS5* and *APETALA3 EpMADS13* are most highly expressed in lip and callus, consistent with current models for lip identity. Six vascular bundles, indicating a stamen-derived origin, lead to the callus, stelidia and stamen. *AGAMOUS* is not expressed in the callus, consistent with its sterilization. Out of three copies of *AGAMOUS* and four copies of *SEPALLATA*, *EpMADS22* and *EpMADS6* are most highly expressed in the stamen. Another copy of *AGAMOUS*, *EpMADS20*, and the single copy of *SEEDSTICK*, *EpMADS23*, are most highly expressed in the stelidia, suggesting *EpMADS22* may be required for fertile stamens.

**Conclusions:**

The median sepal, callus and stelidia of *E. pusilla* appear to be derived from a sepal, a stamen that gained petal identity, and stamens, respectively. Duplications, diversifying selection and changes in spatial expression of different MADS-box genes shaped these organs, enabling the rewardless flowers of *E. pusilla* to mimic an unrelated rewarding flower for pollinator attraction. These genetic changes are not incorporated in current models and urge for a rethinking of the evolution of deceptive flowers.

**Electronic supplementary material:**

The online version of this article (doi:10.1186/s12862-017-0938-7) contains supplementary material, which is available to authorized users.

## Background

Flowering plants interact with a wide range of other organisms including pollinators. Pollinators can either receive nectar, oil, pollen or shelter in return for pollen transfer in a rewarding relationship, or nothing at all in a deceptive relationship [1]. One of the deceptive strategies is mimicry, defined as the close resemblance of one living organism, ‘the mimic’, to another, ‘the model’, leading to misidentification by a third organism, ‘the operator’. Essential for mimicry is the production of a false signal (visual, olfactory and/or tactile) that is used to mislead the operator, resulting in a gain in fitness of the mimic [1]. Mimicry in plants generally serves the purpose of attraction of pollinators to facilitate fertilization. In these cases, an unrewarding plant species mimics traits typical for co-flowering models, such as a specific floral shape, coloration, and presence of nectar guides, glands, trichomes or spurs. In this way, pollinators, that are unable to distinguish the two types of flowers from each other, are fooled [1, 2]. Despite the fact that deceptive pollination evolved in thousands of plant species, most notably orchids [3], the mechanisms by which this deceit evolved are still poorly understood.

Flowers are the main attractors of the majority of angiosperms to gain attention of pollinators. The outer first whorl of a flower is usually made up of sepals that generally serve as protection covering the other floral parts until anthesis. The outer second whorl consists of often-showy petals mainly involved in pollinator attraction. The sepals and petals together enfold the male and female reproductive organs in the inner floral whorls. Over the past decades, evolutionary developmental (evo-devo) studies have yielded many new insights in the role of duplication and neo-functionalization of developmental genes in floral diversification and the evolution of sepals, petals and male and female reproductive organs. These studies helped redefine the evolutionary origin of such organs [4].

Theoretically, an orchid flower can be considered to consist of five whorls of floral organs. Three sepals and three petals are present in the outer two whorls. Three external and three internal stamens and three carpels are present in the three inner whorls (Fig. 5a). Studies of the genetic plant model species *Arabidopsis thaliana* have shown that genes only associated with petals in *A. thaliana* are also expressed in the first floral whorl of petaloid monocots including orchids. Expression of these genes in the first whorl of petaloid monocots plays an important role in the similarity of sepals and petals in lilies, gingers and orchids [5–7]. From an evolutionary perspective, retention of expression of genes associated with petals in the outer floral whorl is considered an ancestral character for angiosperms [8]. In orchid flowers, the median petal, or ‘lip’, is often enlarged and ornamented with a wart-like structure, or ‘callus’. The lip mostly functions as main attractor and landing platform for pollinators. Many hypotheses have been put forward about the evolutionary origin of the lip and its ornaments [9]. Hsu et al. [10] showed that the lip is homologous with true petals but gained an additional function possibly due to the duplication of a complex of modified developmental genes that gained novel expression domains.

A stamen usually consists of a filament and an anther where the pollen are produced. Many lineages in plant families such as buttercups, orchids, penstemons and witch-hazels, not only have fertile stamens but also rudimentary, sterile or abortive stamen-like structures. These structures are generally called staminodes and are often positioned between the fertile stamens and carpels, although they can also occur in other positions [11]. Multiple hypotheses exist about the function of the morphologically very diverse staminodes. In *Aquilegia*, staminodes play a role in protecting the early developing fruits as they usually remain present after pollination long after the other organs have abscised [12]. In other plant genera, staminodes are assumed to mediate pollination. Comparative gene expression and silencing studies showed that staminode identity in *Aquilegia* evolved from a pre-existing stamen identity program. Of the genes involved, one lineage duplicated and one paralog became primarily expressed in the staminodia [11, 12].

Characteristic for orchids is that the male and female reproductive organs are incorporated in a so-called ‘gynostemium’. This structure is thought to result from a fusion of a maximum of six fertile to (partly) sterile stamens and parts of the pistil, in particular the style and stigma. It is a complex organ and the evolutionary origin of its different parts is not yet clear [9, 13, 14]. During the evolution of the orchids over the past 100 million years a reduction in the number of fertile stamens and fusion with the carpels occurred [15–17]. Six fertile stamens, positioned in floral whorls three and four, are commonly present in the closest relatives of the orchids in Asparagales. In the Apostasioideae, the earliest diverging of the five subfamilies of orchids, the number of fertile stamens is reduced to three in the genus *Neuwiedia*, one in floral whorl three and two in whorl four. In the genus *Apostasia*, a staminode develops in floral whorl three or nothing resulting in two fertile stamens [18]. In subfamily Cypripedioideae only two fertile stamens are present. A further reduction into a single fertile stamen in floral whorl three evolved in subfamilies Vanilloideae, Orchidoideae and Epidendroideae [13]. Since the two subfamilies with either three or two fertile stamens are the least diverse, reduction to a single fertile stamen may have contributed to species diversification. The sterile stamens have evolved into many other structures. In the majority of the Epidendroid orchids with a single fertile stamen, the mature gynostemium evolved appendages projecting to the front or side, clearly differentiating from broadened or flattened tissue at the base, that help pollinators to position themselves in the correct way to remove or deposit pollinia, which ensures pollination. The shapes of these appendages differ greatly and different terms are used to describe them, e.g. column wings or ‘stelidia’ [19–21]. The oldest hypothesis postulates that the stelidia are remnants of male reproductive tissue [22, 23] and following this hypothesis, stelidia are interpreted as vestiges of the lateral stamens of the third and fourth floral whorls [24].

### Current models explaining floral organ development

**Fig. 1:**
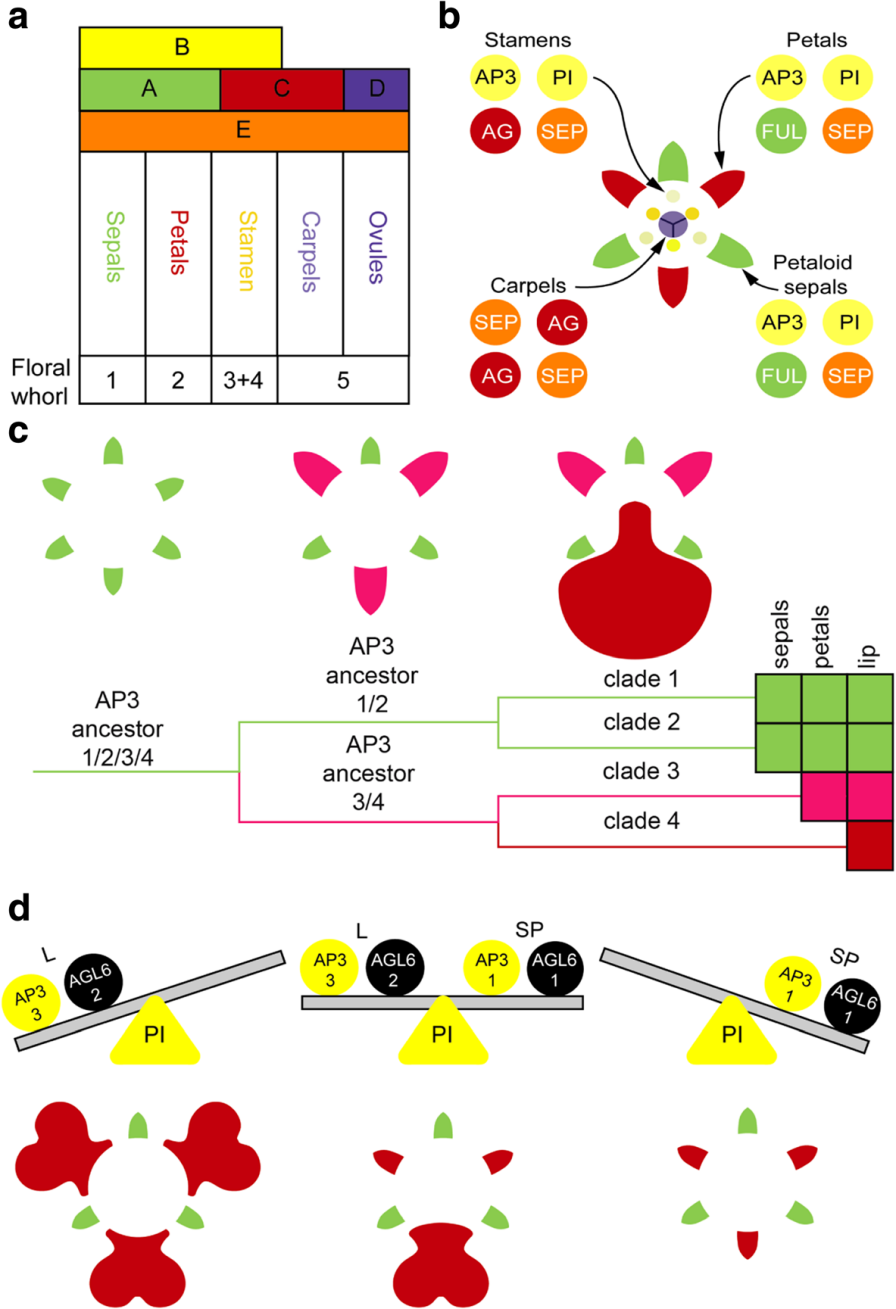
Current models explaining floral organ development. **a** ABCDE model of floral development in petaloid monocots. **b** Floral quartet model. **c** Orchid code and HOT model. **d** Perianth code model [Illustrations by Bas Blankevoort]

The genetic basis of floral organ formation can be explained with various genetic models of MADS-box transcription factors. The core eudicot ‘ABCDE model’ included the A-class gene *APETALA1* (*AP1*), B-class genes *APETALA3* (*AP3*) and *PISTILLATA* (*PI*), C-class gene *AGAMOUS* (*AG*), D-class gene *SEEDSTICK* (*STK*) and E-class gene *SEPALLATA* (*SEP*). This model has been revised for the monocots to reflect two key differences: (i) there are no *AP1* orthologs outside the core eudicots so *FRUITFULL* (*FUL*)-like genes are the closest homologs, and (ii) many monocots have entirely petaloid perianths. Class A + B + E genes specify petaloid sepals, A + B + E control petals, B + C + E determine stamens, C + E specify carpels, and D + E are necessary for ovule development [25–27] (Fig. 1a). As in the core eudicots, these genetic combinations are thought to function as protein complexes, as proposed by Theissen and Saedler [27] in the now well accepted ‘floral quartet model’ (Fig. 1b). For the highly specialized flowers of most orchid lineages, further elaborations have been proposed, including the ‘orchid code’ [28, 29], ‘Homeotic Orchid Tepal’ (HOT) model [30] and ‘Perianth code’ (P-code) [10].

The orchid code and HOT model (Fig. 1c) postulate that the four *AP3* lineages in orchids have experienced sub- and neo-functionalization to give rise to distinct petal and lip identity programs. In addition to original MADS-box genes incorporated in the ABCDE model, several *AGAMOUS-LIKE-6* (*AGL6*) gene copies were recently found to play an important role in orchid flower formation. According to the P-code model (Fig. 1d), there are two MADS-box protein complexes active in orchid flowers, one consisting of a set of *AP3/AGL6/PI* copies, specific for sepal/petal formation, and one consisting of another set of *AP3/AGL6/PI* copies, specific for the formation of the lip. When the ratio of these two complexes is skewed towards the latter, the lip is large. When the ratio is skewed towards the former, intermediate lip-structures are formed [10]. The P-code model has been functionally validated for wild-type *Oncidium* and *Phalaenopsis*, and also for *Oncidium* peloric mutants, in which the two petals are lip-like. The P-code model was also validated in orchids from other subfamilies than the Epidendroideae, to which *Oncidium* and *Phalaenopsis* belong, i.e. Cypripedioideae, Orchidoideae and Vanilloideae, and used to detect gene expression profiles in species with intermediate lip formation [10].

### *Erycina pusilla* as an emergent orchid model: current resources and terminology

MADS-box genes have now been identified for several commercially important orchid genera (e.g. *Cymbidium*, *Dendrobium, Oncidium* and *Phalaenopsis*) [30–32] but long life cycles, large chromosome numbers and complex genomes of these genera hamper functional studies. DNA-mediated transformation can be used to study the function of orchid genes and *E. pusilla*, with its relatively short life cycle, functions as an emergent orchid model species for such studies [33, 34].

**Fig. 2:**
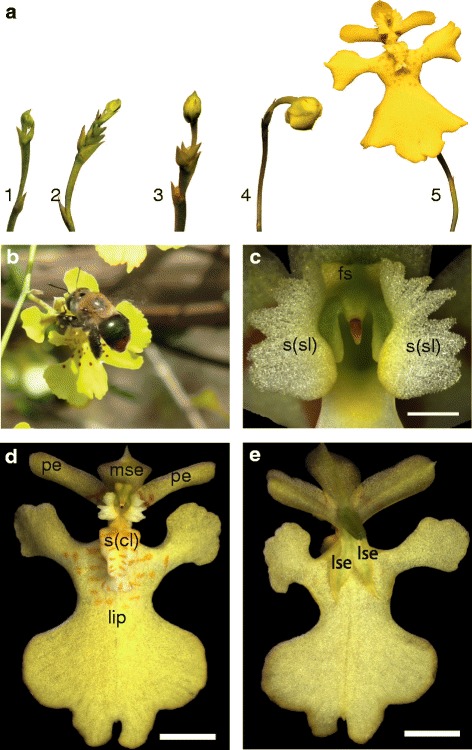
General overview of *E. pusilla* flowers, pollinator and floral parts. **a** Five floral stages of *E. pusilla* [Photo by Rogier van Vugt]. **b** A female *Centris poecila* bee pollinating a flower of *Tolumnia guibertiana*, a close relative of *E. pusilla*, in Cuba [Photo by Angel Vale], showing the function of the stelidia and callus in freshly opened flowers of these orchids, i.e. attraction and providing a holdfast for the pollinator. **c** Frontal view of fully developed stelidia. **d** Adaxial side (with respect to the floral axis) of a flower. **e** Abaxial side (with respect to the floral axis). Abbreviations: s(cl) = callus; lse = lateral sepal; mse = median sepal; pe = petal; s(sl) = stelidium; fs = fertile stamen

*Erycina pusilla* belongs to the Oncidiinae, which is a highly diverse subtribe of meso- and south-American epiphytic orchids in subfamily Epidendroideae [35]. It is a rapidly growing orchid species with a low chromosome number (*n* = 6) and a, for orchids, relatively small sized diploid genome of 1.475 Gb [36, 37]. It can be grown from seed to flowering stage in less than a year [33, 34] and plantlets can be grown without mycorrhizae in test tubes. Flowers develop in a few days in which five distinct floral developmental stages can be observed (Fig. 2a). The species produces deceptive flowers that are self-compatible but incapable of spontaneous self-pollination.

**Fig. 3:**
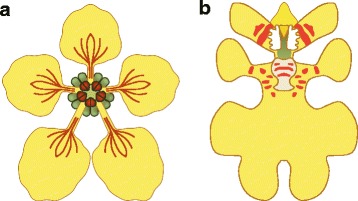
Graphical representation of a flower belonging to (**a**) Malpigiaceae and (**b**) Oncidiinae [Illustrations by Bas Blankevoort]

Oil-collecting *Centris* bees are the main pollinators [38]. The lateral sepals of *E. pusilla* are small and green. The median sepal is larger and more colorful than the lateral sepals. The lip is the largest part of the flower and very different in shape compared to the lateral petals and sepals. On the basal part of the lip or ‘hypochile’, a callus is present that guides pollinators towards the stamen and stigma to either remove or deposit pollinia effectively. The gynostemium is enveloped on both sides by two large, wing-shaped structures that we further refer to as stelidia. During floral visits, *Centris* bees cling to these stelidia and the callus with their forelegs while searching for oils (Fig. 2b). In *E. pusilla* however, these bees are fooled because the flowers employ food deception by Batesian mimicry by resembling flowers of rewarding species of the unrelated Malpighiaceae [38–40]. Flowers of this family have five clawed petals that are often unequal in size. The sepals carry oil glands. It is generally assumed that the enlarged median sepal, incised lip, callus and stelidia of Oncidiinae evolved to mimick the shape of the petals and oil glands of rewarding flowers of Malpighiaceae (Figs. 2b–d and 3) in order to attract oil-collecting bees for pollination [35, 38, 40, 41].

*Agrobacterium*-mediated genetic transformation was recently developed for *E. pusilla* [33] and knockdown of genes is currently being optimized. It is expected that the entire genome will have been analyzed using a combination of next-generation sequencing techniques within the following years. Furthermore, transcriptome data of *E. pusilla* are included in the Orchidstra database [31]. Twenty-eight MADS-box genes from *E. pusilla* have been identified thus far including the most important floral developmental ones [34]. These resources make *E. pusilla* an ideal orchid model for evo-devo studies. Lin et al. [34] published expression data of MADS-box genes isolated from sepals, petals, lip, column and ovary of flowers of *E. pusilla* after anthesis together with a basic phenetic gene lineage analysis.

In this study, we employed a combination of micro-, macromorphological, molecular and phylogenetic techniques to assess the evolutionary origin of the median sepal, callus and stelidia of the flowers of *E. pusilla*. To accomplish this goal, we investigated early and late floral developmental stages with scanning electron microscopy (SEM), light microscopy (LM), 3D-Xray microscopy (micro-CT) and expression (RT-qPCR) of MADS-box genes belonging to six different lineages. In addition, we investigated gene duplication and putative neo-functionalization as indicated by inferred episodes of diversifying selection. Our aim was to test the hypotheses that the median sepal, callus and stelidia are derived from sepals, petals and stamens, respectively, to unravel the genetic basis of the evolution of deceptive flowers.

## Methods

### Plant material and growth conditions

A more than 15 year old inbred line of *E. pusilla* originally collected in Surinam was grown in climate rooms under controlled conditions (7.00–23.00 h light regime), at a temperature of 20 °C and a relative humidity of 50%. The orchids were cultured in vitro under sterile conditions on Phytamax orchid medium with charcoal and banana powder (Sigma-Aldrich) mixed with 4 g/L Gelrite™ (Duchefa) culture medium. Pollinia of flowers from different plants were placed on each other’s stigma after which ovaries developed into fruits. After 18–22 weeks, seeds were ripe and sown into containers with sterile fresh nutrient culture medium. The seeds developed into a new *E. pusilla* flowering plant within 20 weeks.

### Fixation for micromorphology

Flowers and flower buds were fixed with standard formalin-aceto-alcohol (FAA: absolute ethanol, 90%; glacial acetic acid, 5%, formalin; 5% acetic acid) for one hour under vacuum pressure at room temperature and for 16 h at 4 °C on a rotating platform. They were washed once and stored in 70% ethanol until further use.

### SEM

Floral buds at different developmental stages were dissected in 70% ethanol under a Wild M3 stereo microscope (Leica Microsystems AG, Wetzlar, Germany) equipped with a cold-light source (Schott KL1500; Schott-Fostec LLC, Auburn, New York, USA). Subsequently, the material was washed with 70% ethanol and then placed in a mixture (1:1) of 70% ethanol and DMM (dimethoxymethane) for five minutes for dehydration. The material was then transferred to 100% DMM for 20 min and critical point dried using liquid CO_2_ with a Leica EM CPD300 critical point dryer (Leica Microsystems, Wetzlar Germany). The dried samples were mounted on aluminium stubs using Leit-C carbon cement or double-sided carbon tape and coated with Platina-Palladium with a Quorum Q150TS sputtercoater (Quorum Technologies, Laughton, East Sussex, UK). Images were obtained with a JEOL JSM-7600 F Field Emission Scanning Electron Microscope (JEOL Ltd., Tokyo, Japan).

**Fig. 4:**
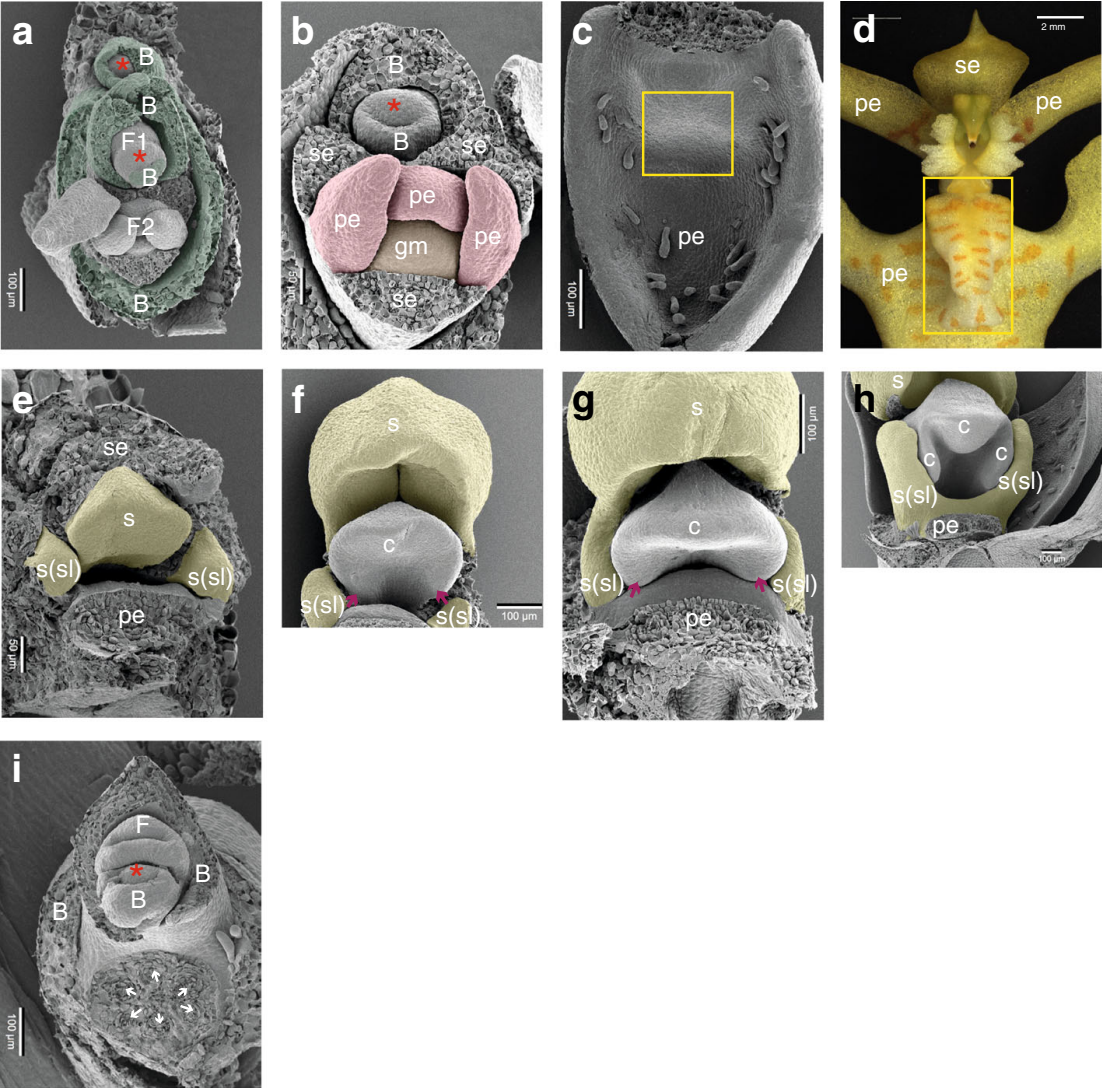
Developing inflorescence of *E. pusilla*. **a** Apical view of a young developing inflorescence. A central meristem is present and below it two flowers are visible, each subtended by a bract. The distal flower (F1) is primordial and the next flower (F2) is somewhat more developed. **b** Apical view of a developing flower in an early developmental stage. The scars of the three removed sepals are visible, two are adaxially (lateral sepals) and one is abaxially (median sepal) situated. More central in the flower, two abaxial-lateral petals and one adaxial developing petal (lip) are present. Most central in the flower is the primordium of the gynostemium. **c**–**d** Developing adaxial petal (lip) with callus (*boxed*). **e**–**h** Successive stages of the development of the gynostemium with the developing fertile stamen central and stelidia laterally. In (**e**), the scar of the removed abaxial sepal is visible. Below the fertile stamen, the scar of the adaxial petal (lip) can be seen. In between the fertile stamen and the adaxial petal (lip), the stigmatic cavity is present. In (**f** and **g**), the two adaxial (lateral) carpels are visible (*arrowed*). In (**h**), the abaxial carpel is incorporated in the stigmatic cavity. **i** Apical view of an inflorescence axis with a removed developing flower. In the upper half of the micrograph, the apex of the axis is visible as well as a flower at very early developmental stage, subtended by a bract. In the lower half, in the scar of the removed developing flower, six vascular bundles are visible (arrowed). Abbreviations: Red asterisk = apical meristem; B = bract; F = flower (primordium); c = carpel; gm = gynostemium; pe = petal; se = sepal; s = fertile stamen; s(sl) = stelidium. Color codes: dark green = bract; red = petals; orange = gynostemium; yellow = androecium

For the images presented in Fig. 4, fixed floral buds were critical point dried using liquid CO_2_ with a CPD 030 critical point dryer (BAL-TEC AG, Balzers, Lichtenstein) and coated with gold with a SPI-ModuleTM Sputter Coater (SPI Supplies, West-Chester, Pennsylvania, USA). Scanning electron microscope (SEM) images were obtained with a Jeol JSM-6360 (JEOL Ltd., Tokyo) at the Laboratory of Plant Conservation and Population Biology (KU Leuven, Belgium).

### 3D-Xray microscopy

Fully grown flowers were infiltrated with 1% phosphotungstic acid (PTA) in 70% ethanol for 7 days in order to increase the contrast [42]. The PTA solution was changed every 1–2 days. The flowers were embedded in 1% low melting point agarose (Promega) prior to scanning. The scans were performed on a Zeiss Xradia 510 Versa 3D X-ray with a Sealed transmission 30–160 kV, max 10 W x-ray sources. Scanning was performed using the following settings: acceleration voltage/power 40 kV/3 W; source current 75 μA; exposure time 2 s; picture per sample 3201; camera binning 2; optical magnification 4 ×, with a pixel size of 3.5 μm. The total exposure time was approximately 3, 2 h. 3D images were stacked and processed with Avizo 3D software version 8.1.

### RNA extraction

For organ dissection, floral buds of *E. pusilla* were collected from floral stages 2 and 4 (Fig. 2a). The earliest floral stage to dissect the different flower parts was at floral stage 2. The lateral sepals, median sepal, petals, lip, callus, stamen and the remaining part of the gynostemium with stelidia but excluding the ovary were dissected (Fig. 2c–e) and collected in individual tubes and immediately frozen on dry ice and stored at –80 °C until RNA extraction. Total RNA was extracted from seven different floral organs of *E. pusilla* using the RNeasy Plant Mini Kit (QIAGEN), following the manufacturer’s protocol. A maximum of 100 mg plant material was placed in a 2.2 ml micro centrifuge tube with 7 mm glass bead. The TissueLyser II (QIAGEN) was used to grind the plant material. The amount of RNA was measured using the NanoVue Plus™ (GE Healthcare Life Sciences) and its integrity was assessed on an Agilent 2100 Bioanalyzer using the Plant RNA nano protocol. RNA samples with an RNA Integrity Number (RIN) < 7 were discarded. RNA was stored at −80 °C until further use. Extracted RNA was treated with DNase I, Amp Grade (Invitrogen 1U/μl) to digest single- and double-stranded DNA following the manufacturer’s protocol.

### cDNA synthesis

cDNA was synthesized with up to 1 μg of DNase-treated RNA using iScript™ cDNA Synthesis Kit (Bio-Rad Laboratories) following the manufacturer’s protocol. A reaction mixture was prepared by addition of 1 μg of RNA, 4 μl 5x iScript reaction mix, 1 μl iScript reverse transcriptase to nuclease-free water up to a total volume of 20 μl. The reaction mixture was incubated at 25 °C for 5 min, 42 °C for 30 min and 85 °C for 5 min using a C1000 Touch™ thermal cycler machine (Bio-Rad). During this reaction, a positive control (CTRL) and no reverse transcriptase (NRT) control were included.

### Primer design

DNA sequences were downloaded from NCBI Genbank and Orchidstra (http://orchidstra2.abrc.sinica.edu.tw). For the MADS-box genes primers were designed on the C-terminal of the DNA sequences to avoid cross –amplification. Beacon Designer™ (Premier Biosoft, http://www.oligoarchitect.com) software was used to design primers (Additional file 1: Tables S1–S2). All primer pairs were screened for their specificity against the Orchidstra database and in a gradient PCR reaction. The reaction mixture (25 μl) contained: 2.5 ng cDNA, 0.2 μM of each primer, 0.1 mM dNTP’s and 0.6 U *Taq* DNA polymerase (QIAGEN) in 1x Coral Load Buffer (QIAGEN). The amplification protocol was as follows: initial denaturation step of 5 min 94 °C followed by 40 cycles of [20 s 94 °C, 20 s <55–65 > °C, 20 s 72 °C], one final amplification step of 7 min 72 °C and ∞ 15 °C. Based on the results of the gradient PCR, the annealing temperature was set to 61.3 °C for the Quantitative Real-time PCR as this value gave the best results. Only when a specific product was detected was the primer pair used for subsequent quantification.

### Reference genes and quantitative real-time PCR

Experimental and computational analyses with LinRegPCR (http://www.hartfaalcentrum.nl, v2015.1) [43, 44] indicate that *E. pusilla Ubiquitin-2*, *Actin*, and *F-box* were stably expressed in the tissues of interest and these genes were chosen as reference genes for the expression assay. Expression of all MADS-box genes was normalized to the geometric mean of these three reference genes.

Quantitative real-time PCR was performed using the CFX384 Touch Real-Time PCR system (Bio-Rad Laboratories). The assays were performed using the iQ™ SYBR® Green Supermix (Bio-Rad Laboratories). The reaction mixture (7 μl) contained: 1x iQ™ SYBR® Green Supermix, 0.2 μM of each primer, 1 ng cDNA template from a specific floral organ (biological triplicate reactions) for each target gene and floral organ for two sets of isolated RNA (six reactions in total). All reactions were performed in Hard-Shell® Thin-Wall 384-Well Skirted PCR Plates (Bio-Rad Laboratories). For each amplicon group, a positive control was included (=CTRL, flower buds from floral stage 1 to 4), a negative control (=NTC, reaction mixture without cDNA) and a no reverse transcriptase treated sample (=NRT, control sample during the cDNA synthesis). For all the qPCR reactions, the amplification protocol was as follows: initial denaturation of 5 min 95 °C followed by; 20 s 95 °C; 30 s 61.3 °C; 30 s 72 °C; plate read, for 50 cycles; then followed by a melting curve analysis of 5 s, 65 °C to 95 °C with steps of 0.2 °C to confirm single amplified products (Additional file 2: Figure S2).

### Normalization, data analysis and statistical analysis

The non-baseline corrected data were exported from the Bio-Rad CFX Manager™ (v3.1) to a spreadsheet. Quantification Amplification results (QAR) were used for analysis with LinRegPCR (v2015.1, dr. J.M. Ruijter). The calculated N_0_-values represented the starting concentration of a sample in fluorescence units. Removal of between-run variation in the multi-plate qPCR experiments was done using Factor qPCR^©^ (v2015.0) [45, 46]. Geometric means of the corrected N_0_-values were calculated from the six samples together, i.e. two biological and three technical replicates. GraphPad Prism version 7.00 (http://www.graphpad.com) was used to perform a Two-Way ANOVA with Sidak’s multiple comparison test to calculate significant differences between the two floral stages 2 and 4, and graphed with Standard Error of Measurement (SEM) error bars. Tukey’s multiple comparisons test was used to compare the means between the floral organs. Variation for the two biological replicates was assessed by tests in triplicate.

### Phylogenetic analyses

Nucleotide sequences of floral developmental genes were downloaded from NCBI GenBank® (Additional file 1: Table S1) and separate data sets were constructed for MADS-box gene classes *FUL-, AP3-, PI-, AG-, STK-, SEP-* and *AGL6-like*. For each gene class, protein-guided codon alignments were constructed by first performing multiple sequence alignments of the protein translations using MAFFT v.7.245 (with the algorithm most suited for proteins with multiple conserved domains, E-INS-I or “oldgenafpair” for backward compatibility), with a maximum of 1000 iterations [47] and then reconciling the nucleotide sequences with their aligned protein translations.

Gene trees were inferred from the codon alignments using PhyML v3.0_360-500M [48] under a GTR + G + I model with six rate classes and with base frequencies, proportion of invariant sites, and γ-shape parameter α estimated using maximum likelihood. Optimal topologies were selected from results obtained by traversing tree space with both nearest neighbor interchange (NNI) and subtree prune and regraft (SPR) branch swap algorithms, ie. PhyML’s “BEST” option. Support values for nodes were computed using approximate likelihood ratio tests (SH-like aLRT, [49]).

To infer where on the gene trees duplications may have occurred the GSDI algorithm [50] was used as implemented in forester V1.038 (https://sites.google.com/site/cmzmasek/home/software/forester). Fully resolved species trees for GSDI testing were constructed based on the current understanding of the phylogeny of the species under study (Additional file 3: Figure S4).

Lastly, to detect lineage-specific excesses of non-synonymous substitutions, BranchSiteREL [51] analyses were performed as implemented in HyPhy [52] on the Datamonkey (http://datamonkey.org) cluster.

## Results

### Ontogeny, macro- and micromorphology of flowers of *E. pusilla*

Floral ontogeny in *E. pusilla* can be divided into two main phases: early and late. Early ontogeny starts from floral initiation (floral stage 1) up to the three-carpel-apex stage (floral stage 2) and late ontogeny starts from the three-carpel-apex stage (floral stage 2) until anthesis (floral stages 3, 4 and 5, Fig. 2a) [53].

**Fig. 5:**
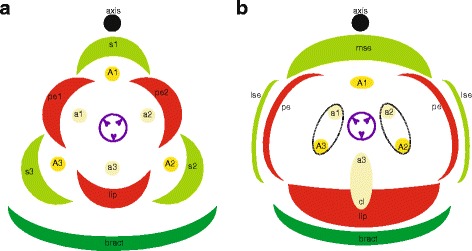
Floral diagrams. **a** A typical monocot flower. **b** A resupinate flower of *E. pusilla*. Abbreviations: s_1–3_ = sepals; p_1–3_ = petals; A_1–3_ = anther in outer floral whorl; a_1–3_ = anther in inner floral whorl; lse = lateral sepal; mse = median sepal; pe = petal; cl = callus. Color codes: black interrupted = stelidia and callus on lip; purple = gynoecium [Illustrations by Erik-Jan Bosch]

The inflorescence of *E. pusilla* is branched and multiple flowers develop in succession (Fig. 2a). Up to floral stage 1, the perianth is formed following a classic monocot developmental pattern (Fig. 5a) [54] in which the sepals are among the first organs to become visible, followed by the petals. The position of the two abaxial petals is slightly shifted laterally (Fig. 4a). Stamen and carpel primordial are not visible in the course of the early phase, but instead a single massive primordium is present from which the gynostemium will develop (Fig. 4b).

On the hypochile of the lip a callus is formed from floral stage 2 onwards (Fig. 4c–d). The fertile stamen differentiates after floral stage 1. The stelidia appear at each side of the gynostemium (Fig. 4e–h) from where they elongate and start forming wing-like appendices (Fig. 2e). The abaxial carpel is incorporated in the stigmatic cavity, which forms a compound structure with the fertile stamen (Fig. 4h). The three-carpel-apex stage is clearly visible in floral stage 2. At this stage the six staminal vascular bundles can also be observed just above the inferior ovary (Fig. 4i). In floral stage 3, no new organs are formed, but in floral stage 4 (Fig. 2a) the mature flower becomes resupinate (Fig. 5b). The terms adaxial and abaxial are used here to indicate the position of the distinct floral parts with respect to the inflorescence axis (Fig. 4a–b), thereby taking the position of the primordia of the floral organs as a reference. For example, with respect to the inflorescence axis, the lip is the adaxial petal, which by resupination becomes the lowermost part of the flower.

**Fig. 6:**
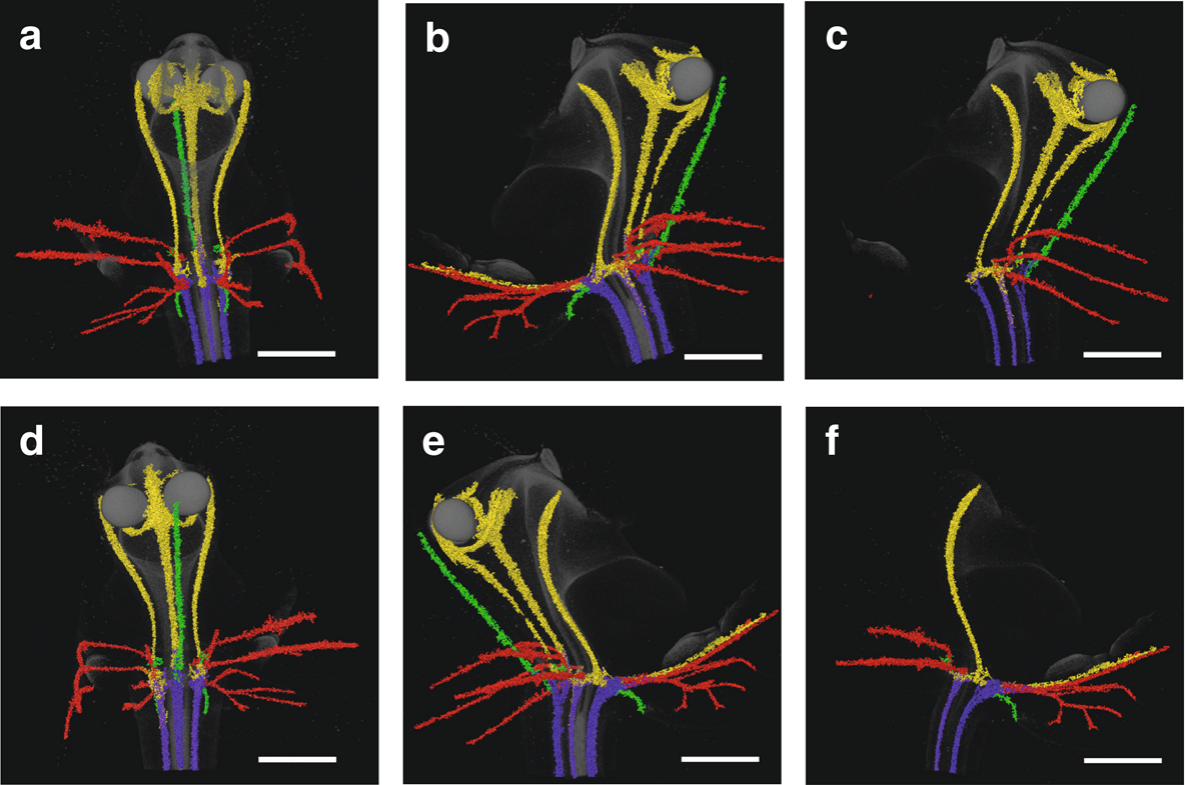
Vascular bundle patterns of *E. pusilla.* **a** Frontal view of a 3D X-ray macroscopical reconstruction of the vascular bundle patterns in a mature flower of *E. pusilla.* **b** Successive clockwise turn of 45°. **c** Simplified version of (**b**). **d** Successive clockwise turn of 90°. **e** Successive clockwise turn of 135°. **f** Simplified version of (**e**). Color codes: green = vascular bundles in sepals; red = vascular bundles in petals; purple = vascular bundles in gynoecium; yellow = vascular bundles in androecium. Scale bar = 1 mm


Using micro-CT scanning, vascular bundles were observed in a fully-grown floral stage 5 flower (Fig. 6a–f and Additional file 4: Movie S1). In the inferior ovary six vascular bundles could be discerned, indicated in purple. Three of these vascular bundles, indicated in green, run to the adaxial (median) sepal and abaxial (lateral) sepals, respectively. Three main groups of vascular bundles, indicated in red, run towards the petals including the lip, where they split up. Four vascular bundles (indicated in yellow) are present; one bundle, already split into two at the base, runs to the fertile stamen, where it splits up further towards the two pollinia (Fig. 6a–e); two vascular bundles, originated from two pairs, run up into the stelidia (Fig. 6b–c; e–f) and one vascular bundle runs all the way up into the callus of the lip (Fig. 6b; e–f). When following the yellow vascular bundles downwards, they connect in a plexus situated on top of the inferior ovary with the rest of the vascular system of the flower.

Additional file 4: Movie S1. Animation of the 3D visualization as depicted in Fig. 6. (MPG 60302 kb)


**Fig. 7:**
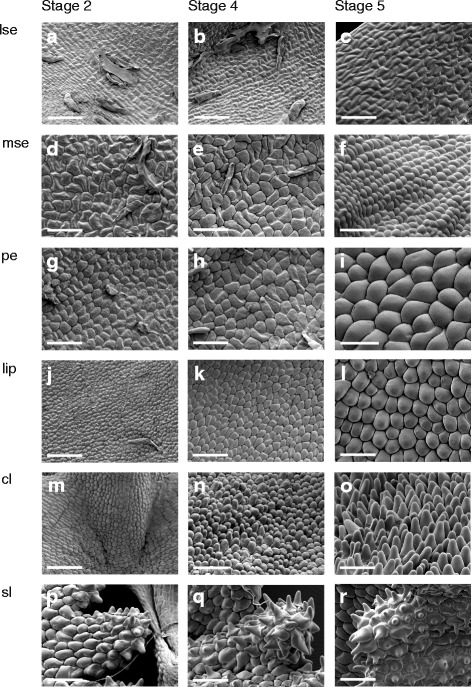
Micromorphology of the epidermal cells on the adaxial side of a flower of *E. pusilla*. The three columns represent, from left to right, floral stage 2, 4 and 5 of the floral organs. Epidermal cells of (**a**–**c**) lateral sepal, (**d**–**f**) median sepal, (**g**–**i**) petal, (**j**–**l**) lip, (**m**–**o**) callus on lip and (**p**–**r**) stelidia. Scale bar = 100 μm. Abbreviations: lse = lateral sepal; mse = median sepal; pe = petal; cl = callus; sl = stelidia

Throughout late ontogeny, epidermal cells in all floral organs remained relatively undifferentiated and only expanded in size. Epidermal cells on the abaxial side of floral organs were mostly similar to the cells on the adaxial side, but more convex shaped (Additional file 5: Figure S1). Epidermal cells of the lateral sepals were irregular, flattened and rectangular shaped and longitudinally orientated from the base to the apex (Fig. 7a–c). Epidermal cells of the median sepal, as well as of the petals and the lip, develop from irregularly flattened shaped cells at floral stage 2, to a more convex shape in floral stage 5 (Fig. 7d–l). Epidermal cells of the callus develop from convex shaped cells in floral stage 2 to cells with a more conical shape in floral stage 5 (Fig. 7m–o). Epidermal cells of the stelidia become convex shaped during floral stage 2 and develop papillae on their apices during floral stage 5 (Fig. 7p–r).

### Duplications, diversifying evolution and expression of eighteen MADS-box genes in selected floral organs of *E. pusilla* in two developmental stages

#### FUL-, SEP- and AGL6-like genes

The closest homologs of the *Arabidopsis* A class gene *APETALA1* in *E. pusilla* are the three *FUL*-like genes copies *EpMADS10*, *11* and *12*. Our phylogenetic analyses reconstructed three orchid clades of *FUL*-like genes, containing the three copies present in the genome of *E. pusilla* (Additional file 6: Figure S5a), which was consistent with previous studies [55]. Diversifying selection was detected along the branch following the gene duplication leading to *EpMADS10*. The three *FUL-*like gene copies were expressed in all floral organs of *E. pusilla* but at low levels only (Additional file 7: Figure S3). During development, expression generally decreased in most floral organs for *EpMADS10* and *11* whereas it generally increased for the majority of floral organs for *EpMADS12* (Additional file 7: Figure S3 and Additional file 1: Table S3).

Four *SEP*-like orchid clades were retrieved (Additional file 6: Figure S5f), encompassing the four copies of *E. pusilla*, consistent with previous studies [55, 56]. The branch leading to the duplication that gave rise to *EpMADS6* and *EpMADS7* shows evidence of diversifying selection. *EpMADS6, 7, 8* and *9* were expressed in all floral organs at varying levels. *EpMADS6* was mainly expressed in the fertile stamen, a statistically significant difference as compared to the other six floral organs (Additional file 7: Figure S3 and Additional file 1: Table S3).

**Fig. 8:**
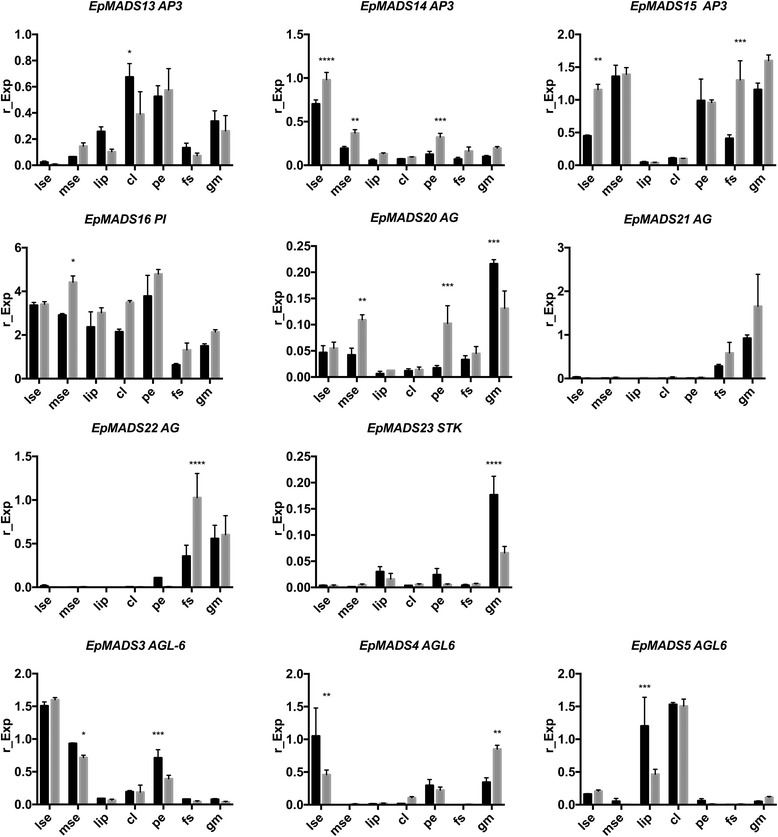
Floral organ specific expression levels of selected MADS-box gene copies in *E. pusilla. AP3* (top row), *PI* (second row), *AG* (second and third row), *STK* (second row), *ALG6* (third row). RNA was extracted from seven different floral organs during two stages of development of *E.* pusilla and used for cDNA synthesis. Expression of the MADS-box genes was normalized to the geometric mean of three reference genes *Actin*, *UBI2* and *Fbox*. Each column shows the relative expression of 20 floral organs in two cDNA pools (10 floral organs per isolation), both tested in triplicate. Abbreviations: lse = lateral sepal; mse = median sepal; cl = callus; pe = petal; fs = fertile stamen; gm = gynostemium. Dark grey = floral stage 2 and light grey = floral stage 4. Y-axis: relative gene expression. The *error bars* represent the Standard Error of Mean. *P*-value style: GP: >0.05 (ns), <0.05 (*), <0.01 (**), <0.001 (***), <0.0001 (****)

Three *AGL6* orchid clades, also found by Hsu et al. [10] were retrieved, containing the three different copies present in the *E. pusilla* genome (Additional file 6: Figure S5g). Evidence for a moderate degree of diversifying selection could be detected on the branch leading to *EpMADS4.* The three different copies of *AGL6*-genes were not expressed in all floral organs and the level of expression also varied. *EpMADS3* was most highly expressed in the sepals and petals. *EpMADS4* was more highly expressed in the lateral sepals as compared with the median sepal, petals and lip. *EpMADS5* was mainly expressed in the lip and callus (Fig. 8).

#### AP3-like and PI-like genes

**Fig. 9:**
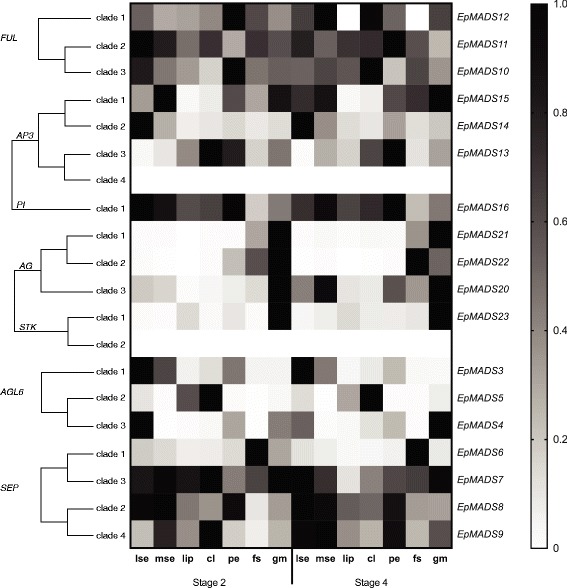
Heat map representation of MADS-box gene expression in *E. pusilla.* The *FUL-, AP3-, PI-, AG-, STK-, SEP-* and *ALG6-* like copies were retrieved from different gene lineage clades during two stages of floral development. Expression of the MADS-box genes was normalised to the geometric mean of three reference genes *Actin*, *UBI2* and *Fbox*. The relative gene expression was normalised with the CTRL sample (= flower buds from floral stages 1-4). The scales for each gene and developmental stage are independent of each other and set to 1 for the highest value. Abbreviations: lse = lateral sepal; mse = median sepal; cl = callus; pe = petal; fs = fertile stamen; gm = gynostemium

Initial phylogenetic analyses reconstructed the main duplication between the *AP3* and *PI* genes also found in many other studies [10, 30, 57] so two separate gene trees were retrieved for each lineage (Additional file 6: Figure S5b–c). Four orchid *AP3*-clades and three *PI*-clades were identified in these analyses. The three copies of *AP3* and a single copy of *PI* present in the genome of *E. pusilla* were placed in *AP3*-clades 1, 2 and 3 and *PI*-clade 2, respectively. No evidence for diversifying selection could be detected along the branches leading to the *PI*-clade containing *EpMADS16* but evidence for diversifying selection along the branch in the *AP3-*1 clade encompassing *EpMADS15* was found. *AP3*-like gene copy *EpMADS14* was most highly expressed in the lateral sepals. *AP3*-like gene copy *EpMADS13* was more highly expressed in the lip and callus than in the sepals and petals (Fig. 8). The *PI*-like gene *EpMADS16* was more highly expressed in the first four floral whorls in both floral stages (Figs. 8 and 9).

#### AG- and STK-like genes

Three orchid *AG*-clades and two *STK*-clades were identified in the phylogenetic analyses (Additional file 6: Figure S5d–e). *EpMADS20, 21 and 22* were placed in *AG-*clades 3, 1 and 2, respectively, and *EpMADS23* was placed in *STK-*clade 1, as also found by Lin et al. [34]. No evidence for diversifying selection in the branches supporting the three orchid *AG*-clades and *STK*-clade containing copies present in the genome of *E. pusilla* could be detected. *AG*-like gene copy *EpMADS20* was most highly expressed in the stelidia, whereas *EpMADS22* was most highly expressed in the stamen as compared with all other floral organs analyzed (Fig. 8). No expression of *AG*-like genes could be detected in the callus. *STK*-like gene copy *EpMADS23* was most highly expressed in the stelidia as compared with all other floral organs analyzed (Figs. 8 and 9).

## Discussion

### Homology of the median sepal of *E. pusilla*

The floral ontogenetic observations and vascularization patterns indicate that the median sepal is derived from the first floral whorl. In contrast, the presence of convex epidermal cells suggests a petaloid origin [58]. The *AGL6* and *AP3* copies *EpMADS3* and *EpMADS15,* members of the sepal/petal-complex of the P-code model, were most highly expressed in the median sepal, lateral sepal and petal. A possible correlation between expression and petaloidy was found for *AGL6* and *AP3* copies *EpMADS4* and *EpMADS14.* These two genes were lowly expressed in the median sepal, lip and petal as compared with the lateral sepal. Additional functional studies are needed to show whether loss of function of *EpMADS4* and *EpMADS14* is linked to sepal morphology in *E. pusilla* and other species that also possess a petaloid median sepal. The *AGL6* gene copy *EpMADS4* copy showed evidence of diversifying evolution. Lin et al. [34] identified fifteen motifs in the MIKC-type MADS-box proteins of *E. pusilla*. Two differences can be noticed within the K-region and C-terminal-region of *AP3* and *AGL6* genes of *E. pusilla*: (i) *AP3 EpMADS14* is missing motif 11, while the other B-class genes all contain motif 11. *AGL6 EpMADS4* also contains motif 11, while the other *AGL6* gene copies lack this motif; (ii) *AGL6 EpMADS4* is missing motif 6 whereas all the other *AGL6* gene copies contain motif 6. The differences found may contribute to the morphological differences between the median and lateral sepals *of E. pusilla*.

### Homology of the lip and callus of *E. pusilla*

The convex shaped epidermal cells on the lip and conical shaped epidermal cells on the callus are indicative of a petaloid function [58]. The *FUL*-like gene copy *EpMADS12*, *AP3-*like *EpMADS13* and *AGL6*-like *EpMADS5* are most highly expressed in lip and callus, further confirming a lip identity based on the ABCDE, floral quartet and P-code models, that dictate joint expression of A, B, E and *AGL6*-like genes in the petals and lip, respectively. According to these models, B, C and E class genes should be expressed in stamens but no evidence of expression of C class genes was found in the lip or callus. Notwithstanding, the possible staminal origin of the callus is supported by multiple lines of evidence. First of all, the ontogeny and function of the lip of *E. pusilla* are very different as compared with the ontogeny and function of the callus. The lip is formed from floral stage 1 onwards, mainly acts as a long distance attraction and functions as a soft landing platform for pollinating bees. The callus is formed from floral stage 2 onwards and functions as short distance attraction by offering a sturdy holdfast to pollinators. This is in line with Carlquist [59], who states that different vascularization patterns are driven by different functional needs. Many Oncidiinae have a callus on the lip and in some of these species, the callus produces oil, making the functions of the lip and the callus even more distinct. Flowers with an oil-producing callus evolved twice in unrelated clades from species with non-rewarding flowers according to the molecular phylogeny of the Oncidiinae as presented in Pridgeon et al. [38]. One of the two rewarding clades, i.e. the one containing the genus *Gomesa*, is the sister group of the *Erycina* clade, showing that changes between an oil-producing and a non-rewarding callus occur quite easily in this group of orchids. This suggests that evolution towards oil production is correlated with increased venation as also stated by Carlquist [59]. We argue, however, that the venation in the callus is not only driven by functional needs but that the venation pattern is also informative regarding the evolutionary origin of the callus, as the callus of *E. pusilla* is connected with only one of the six original staminal bundles, physically distinct from the two adjacent vascular bundles leading to the lip. We consider this indicative of a possible staminal origin of the callus because of the occasional appearance of an infertile staminodial structure at this particular position, the inner adaxial stamen (a3), in teratologous orchid flowers [60]. Terata of monandrous orchids with both stelidia carrying an additional anther on their tip next to the anther on the apex of the gynostemium, such as *Bulbophyllum triandrum* and *Prostechea cochleata* var. *triandrum*, are commonly seen as support for a staminal origin of stelidia. Similarly, mutants in *Dactylorhiza* with a staminodial structure on their lip [60] could be interpreted as support for a staminal origin of the callus. Alternatively, these phenotypes could be caused by ectopic C gene expression that is transforming petal into stamen tissue. Homeotic transformation is not necessarily indicative of derivation. According to Carlquist [59] data from teratology are therefore not useful for studying the evolution of flowers. This publication was written at a time that experimental mutants could not yet be made though. Ongoing work on B- and C- class homeotic mutants in the established plant models *Arabidopsis, Antirrhinum* and *Petunia* shows how much can be gained from teratology. We hope that these mutants can be created in emerging orchid models such as *E. pusilla* in the future to provide more evidence for the evolutionary origin of the callus on the lip.

### Homology of the stamen and stelidia of *E. pusilla*

Five vascular bundles, indicating a stamen-derived origin, lead to the stamen and stelidia. Our observations concur with those of Swamy [24] who showed that the ovary is traversed by multiple vascular bundles in monandrous orchids. He visualized ‘compound’ bundles of staminal origin in the ovary of a species of *Dendrobium* and discovered vascularizing bundles in the stelidia. In several other plant families, e.g. Brassicaceae (*Arabidopsis*), Commelinaceae (*Tradescantia*), and Cyperaceae (*Cyperus*), it has been shown that vascular bundles of different organs originate in the developing organs and grow towards the stele rather than being branched from the stele [61–64]. Based on Fig. 6 and Additional file 4: Movie S1, we hypothesize that especially the staminal vascular bundles are connected in a similar way to the rest of the vascular system. Of the three copies of *AG* and four copies of *SEP*, *EpMADS22* and *EpMADS6* were found to be highest expressed in the stamen. Another copy of *AG*, *EpMADS20*, and the single copy of *STK*, *EpMADS23*, were found to be most highly expressed in the stelidia, suggesting that *EpMADS23* expression may be correlated with sterility.

### Implications for current floral models

**Fig. 10:**
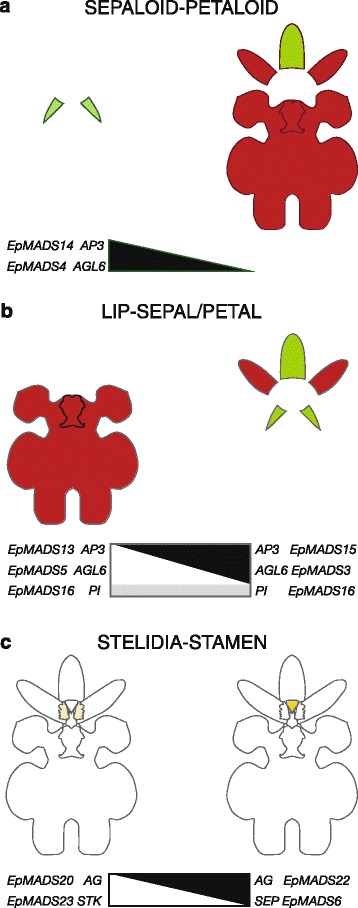
Summary of expression of MADS-box genes involved in the differentiation of selected floral organs of *E. pusilla*. **a** Expression of *EpMADS4*/*14* (in *black*) correlating with a sepaloid-petaloid identity is high in the lateral sepals (*left side*) but low in the remainder of the perianth (*right side*), **b** Expression of the lip complex *EpMADS5/13/16* (in *white*/*grey*)) correlating with a lip identity is high in in the lip and callus (*left side*) but low in the remainder of the perianth (*right side*). Expression of the sepal/petal-complex *EpMADS3/15/16* (in *black*/*grey*) correlating with a sepal and petal identity is low in the lip (*left side*) but high in the sepals and petals (*right side*), **c** Expression of *EpMADS20*/*23* (in *white*) correlating with a stelidia-stamen identity is high in the stelidia (*left side*) but low in the stamen (*right side*). Expression of *EpMADS6/22* (in black) is low in the stelidia (left side) but high in the stamen (*right side*)

The ABCDE, orchid code, HOT and P-code models do not explain the morphological difference between median and lateral sepals as present in orchid species such as *E. pusilla*. Our results show that a differentiation between the sepaloid lateral sepals and petaloid median sepal of *E. pusilla* is correlated with a significant reduction of expression of *AP3*-like *EpMADS14* and *ALG6*-like *EpMADS4* in all petaloid organs (Fig. 10a).

**Fig. 11:**
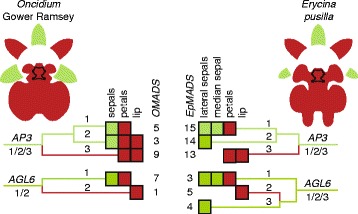
Oncidiinae model summarizing expression of MADS-box genes involved in the differentiation of the perianth of *Oncidium* Gower Ramsey (*left*) and *E. pusilla* (*right*). Clade 1 *AP3*-like *OMADS5* and *EpMADS15* and clade 1 *AGL6*-like genes *OMADS7* and *EpMADS3* are expressed in the sepals and petals of both species. Clade 2 *AP3*-like *OMADS3* is expressed in the entire perianth of *O.* Gower Ramsey whereas *EpMADS14* is only expressed in the lateral sepals of *E. pusilla*. Clade 2 *AGL6*-like genes *OMADS1* and *EpMADS5* are expressed in the lip only of both species. Clade 3 *AP3*-like *OMADS9* and *EpMADS13* are expressed in the petals and lip of both species. Clade 3 *AGL6*-like gene *EpMADS4* is only expressed in the lateral sepals of *E. pusilla* [Illustrations by Bas Blankevoort]

The P-code model explains the development of the lip of *E. pusilla* as the SP-complex (*AP3-*like *EpMADS15/AGL6-*like *EpMADS3/PI-*like *EpMADS16)* was found to be most highly expressed in the sepals and petals, whereas the L-complex (*AP3-*like *EpMADS13/AGL6-*like *EpMADS5/PI-*like *EpMADS16)* was found to be most highly expressed in the lip (Fig. 10b). However, the model does not yet account for the development of the callus and the high expression of *AGL6-*like *EpMADS5* in this particular organ. To incorporate all new evidence found for the evolution and development of first and second floral whorl organs, we propose an Oncidiinae model (Fig. 11), summarizing the gene expression data presented in this study for *E. pusilla* and earlier studies carried out on *Oncidium* Gower Ramsey [10] [Illustrations by Bas Blankevoort].

All four MADS-box B class gene copies were found to be expressed in the fertile stamen of *E. pusilla*. In addition, *AG*-like *EpMADS22* and *SEP*-like *EpMADS6* were most highly expressed in this floral organ, confirming a stamen identity as predicted by the ABCDE model. The high expression of *AG*-like *EpMADS20* and *STK*-like *EpMADS23* in the stelidia cannot be explained with the ABCDE model. All current orchid floral models only describe evolution and development of the first and second whorl floral organs. We found evidence for differential gene expression in organs in the third and fourth floral whorl, i.e. the stamen and stelidia (Fig. 10c), and this argues for the development of additional models.

## Conclusions

After examining vascularization, macro- and micromorpology, gene duplications, diversifying evolution and expression of different MADS-box genes in selected floral organs in two developmental stages, it can be concluded that: (i) the median sepal obtained a petal-identity, thus representing a particular character state of the character ‘sepal’, (ii) that the lip was derived from a petal but the callus from a stamen that gained petal identity, and (iii) the stelidia evolved from stamens. Duplications, diversifying selection and changes in spatial expressions of *AP3 EpMADS14* and *AGL6 EpMADS4* may have contributed to an increase of petaloidy of the median sepal. The same can be applied to *AP3 EpMADS13* and *AGL6 EpMADS5* in the lip and callus. Differential expression of *AG* copies *EpMADS20* and *EpMADS22*, *STK* copy *EpMADS23* and *SEP* copy *EpMADS6* appear to be associated with the evolution of the stamen and stelidia, respectively.

The evolutionary origin of the median sepal, callus and stelidia of *E. pusilla* cannot be explained with any of the currently existing floral developmental models. Therefore, new models, like our Oncidiinae model, need to be developed to summarize MADS-box gene expression in more complex floral organs. Such models need validation by functional analyses. The genetic mechanisms discovered in this study ultimately contributed to the evolution of a deceptive orchid flower mimicking the morphologies of rewarding Malpighiaceae flowers. This mimicry enabled flowers of *E. pusilla,* and many other species in the highly diverse Oncidiinae, to successfully attract *Centris* bees for pollination, often, as is the case for *E. pusilla*, without offering a reward. Pollination by deceit is one of the most striking adaptations of orchids to pollinators. It is estimated that approximately a third of all orchid species employ deceit pollination, and that food mimicry is the most common type. Deceptive pollination is hypothesized to be correlated with species diversification as subtle changes in floral morphology can attract different pollinators and eventually lead to reproductive isolation. It was recently discovered that deceptive pollination augmented orchid diversity, not by accelerating speciation but by adding more species at roughly the same rate through time [17]. Ongoing research on the genomics of *E. pusilla* and other emergent plant models will shed more light on the role that key developmental genes played in the evolution of deceptive flowers.

## Additional files

Additional file 1: Table S1. List of sequences used in the alignments and phylogenetic analyses. **Table S2.** Transcript primer sequences and amplicon characteristics used for quantitative real-time PCR validation of the expression profiles of eighteen MADS-box transcripts following MIQE guidelines [65]. **Table S3.** Difference in MADS-box gene expression between floral organs; variance analysis of measures using Tukey multicomparisons test. *P*-value style: GP: >0.05 (ns), <0.05 (*), <0.01 (**), <0.001 (***), <0.0001 (****). Abbreviations: lse = lateral sepal, mse = median sepal, cl = callus, pe = petal, fs = fertile stamen and gm = gynostemium. (DOCX 110 kb)

Additional file 2: Figure S2. Melting curve analysis of all primer pairs used in this study performed at the end of the PCR cycles to confirm the specificity of primer annealing. (PDF 9997 kb)

Additional file 3: Figure S4. Species phylogeny compiled based on Topik et al. [66], Biswal et al. [67], Takamiya et al. [68] and Chase et al. [69] for (a) *FUL*-, (b) *AP3*-, (c) *PI*- (d) *AG*- and *STK*-, (e) *SEP*- and (f) *AGL6*-like MADS-box gene lineage trees. (ZIP 584 kb)

Additional file 5: Figure S1. Scanning electron micrographs of epidermal cells on the abaxial side of an *E. pusilla* flower. The three columns represent, from left to right, stage 2, 4 and 5 floral organs. Epidermal cells of (a–c) lateral sepal, (d–f) median sepal, (g–i) petal and (j–l) lip. Scale bar = 100 μm. Abbreviations: lse = lateral sepal; mse = median sepal; pe = petal. (PDF 19591 kb)

Additional file 6: Figure S5. MADS-box gene lineage trees. (a) *FUL*-, (b) *AP3*-, (c) *PI*-, (d) *AG*-, (e) *STK*-, (f) *SEP*- and (g) *AGL6*-like trees. Color codes: green node = speciation event; red node = duplication event. Branches are colored along a gradient between blue and red, in proportion to the value of omega (dN/dS) for the third (i.e. the highest) rate class in the BranchSiteREL analysis. Hence, blue and red branches may be interpreted as suggesting, respectively, stabilizing and diversifying selection. Purple branches implicate a moderate level of diversifying selection. (ZIP 1121 kb)

Additional file 7: Figure S3. Floral organ specific expression levels of *FUL EpMADS10, EpMADS11* and *EpMADS12* and *SEP EpMADS6*, *EpMADS7, EpMADS8* and *EpMADS9*. RNA was extracted from seven different floral organs during two stages of development of *E. pusilla* and used for cDNA synthesis. Expression of the MADS-box genes was normalized to the geometric mean of three reference genes *Actin*, *UBI2* and *Fbox*. Each column shows the relative expression of 20 floral organs in two cDNA pools (10 floral organs per isolation), both tested in triplicate. Abbreviations: lse = lateral sepal; mse = median sepal; cl = callus; pe = petal; fs = fertile stamen; gm = gynostemium. Dark grey = floral stage 2 and light grey = floral stage 4. Y-axis: relative gene expression. The error bars represent the Standard Error of Mean. *P*-value style: GP: >0.05 (ns), <0.05 (*), <0.01 (**), <0.001 (***), <0.0001 (****). (TIFF 3032 kb)

## Abbreviations

*AG*: *AGAMOUS*
*AGL6*: *AGAMOUS-LIKE-6*
*AP3*: *APETALA3*
cl: callus
fs: fertile stamen
*FUL*: *FRUITFULL*
gm: gynostemium
L-complex: Lip complex
LM: Light microscopy
lse: lateral sepal
micro-CT: 3D-Xray microscopy
mse: median sepal
P-code: Perianth code
pe: petal
*PI*: *PISTILLATA*
SEM: Scanning electron microscopy
*SEP*: *SEPALLATA*
SP-complex: Sepal/petal-complex
*STK*: *SEEDSTICK*
