# Supplementary material for: Exploring the evolutionary origin of floral organs of Erycina pusilla, an emerging orchid model system
Source: BMC Evol Biol. 2017 Mar 23;17:89. doi: 10.1186/s12862-017-0938-7 (PMC5364718; doi:10.1186/s12862-017-0938-7)
Supplement: Supplementary file 2 — List of sequences used in the alignments and phylogenetic analyses. Table S2. Transcript primer sequences and amplicon characteristics used for quantitative real-time PCR validation of the expression profiles of eighteen MADS-box transcripts following MIQE guidelines [65]. Table S3. Difference in MADS-box gene expression between floral organs; variance analysis of measures using Tukey multicomparisons test. P-value style: GP: >0.05 (ns), <0.05 (*), <0.01 (**), <0.001 (***), <0.0001 (****). Abbreviations: lse = lateral sepal, mse = median sepal, cl = callus, pe = petal, fs = fertile stamen and gm = gynostemium. (DOCX 110 kb) [file 12862_2017_938_MOESM1_ESM.docx]

**Additional file 1**

**Table S1**: List of sequences used in the alignments and phylogenetic analyses. All sequences listed were downloaded from NCBI GenBank.

| **Class** | **Name** | **Species** | **Accession** |
| --- | --- | --- | --- |
| A | *AlFL* | *Allium sp. AL-2003* | AY306138 |
| A | *FL1* | *Alpinia oblongifolia* | EF521814 |
| A | *AlsSQa* | *Alstroemeria ligtu subsp.* | AB618669 |
| A | *AlsSQb* | *Alstroemeria ligtu subsp.* | AB618670 |
| A | *FL4* | *Aristolochia salvadorensis* | KF500109 |
| A | *FL1* | *Aristolochia salvadorensis* | KF500108 |
| A | *FL3* | *Aristolochia salvadorensis* | KF500111 |
| A | *FL2* | *Aristolochia salvadorensis* | KF500110 |
| A | *FL2* | *Aristolochia trilobata* | KF500106 |
| A | *FL1* | *Aristolochia trilobata* | KF500112 |
| A | *FL1* | *Asarum caudatum* | KF500105 |
| A | *FL1* | *Asarum europaeum* | KF500104 |
| A | *FL3* | *Asarum europaeum* | KF500107 |
| A | *Kcap1b* | *Crocus sativus* | AY337929 |
| A | *Kcap1a* | *Crocus sativus* | AY337928 |
| A | *Kcap1c* | *Crocus sativus* | AY337930 |
| A | *AP1* | *Cymbidium ensifolium* | JX255735 |
| A | *MADS1* | *Cymbidium faberi* | KC148540 |
| A | *AP11* | *Cymbidium faberi* | JQ031272 |
| A | *DthyrFL2* | *Dendrobium thyrsiflorum* | AY927237 |
| A | *SQUA1* | *Elaeis guineensis* | AF411840 |
| A | *EgMADS17* | *Elaeis guineensis* | DQ333324 |
| A | *EpMADS10* | *Erycina pusilla* | KJ002735 |
| A | *EpMADS11* | *Erycina pusilla* | KJ002736 |
| A | *EpMADS12* | *Erycina pusilla* | KJ002737 |
| A | *MADS5* | *Lilium longiflorum subsp.* | HQ149331 |
| A | *AP1* | *Magnolia grandiflora* | AY821777 |
| A | *MpMADS15* | *Magnolia praecocissima* | AB050657 |
| A | *AP1* | *Narcissus tazetta var.* | JN704304 |
| A | *MADS10* | *Oncidium* Gower-Ramsey | HM140846 |
| A | *MADS15* | *Oryza sativa* | AF058698 |
| A | *MADS14* | *Oryza sativa* | AF058697 |
| A | *ORAP13* | *Phalaenopsis amabilis* | DQ104327 |
| A | *ORAP11* | *Phalaenopsis amabilis* | DQ104328 |
| A | *FL1* | *Saruma henryi* | KF500102 |
| A | *MADS1* | *Sedirea japonica* | JQ776636 |
| A | *TvFL1* | *Tradescantia virginiana* | AY306190 |
| A | *TGSQB* | *Tulipa gesneriana* | AB472011 |
| A | *ZAP1* | *Zea mays* | NM001111863 |
| A | *MADS3* | *Zea mays* | NM001111457 |
| A | *m15* | *Zea mays* | AJ430632 |
| A | *m4* | *Zea mays* | NM001111681 |
| A | *m28* | *Zea mays* | AJ430695 |
| B | *ApDEF* | *Agapanthus praecox* | AB177941 |
| B | *MADS5* | *Alpinia hainanensis* | AY621154 |
| B | *AmAP3* | *Amborella trichopoda* | AB154845 |
| B | *AODEF* | *Asparagus officinalis* | AB094964 |
| B | *BsAP3* | *Brasenia schreberi* | AB158355 |
| B | *CCDEF* | *Commelina communis* | AB177808 |
| B | *AP3* | *Cymbidium ensifolium* | JQ326261 |
| B | *AP3* | *Cymbidium ensifolium* | JQ326260 |
| B | *AP3* | *Cymbidium ensifolium* | JN613151 |
| B | *AP3* | *Cymbidium faberi* | HM208536 |
| B | *DEF* | *Cymbidium faberi* | HM208535 |
| B | *AP3* | *Cymbidium goeringii* | HM106983 |
| B | *DEF* | *Cymbidium goeringii* | HM106982 |
| B | *AP3A* | *Dendrobium crumenatum* | DQ119838 |
| B | *AP3B* | *Dendrobium crumenatum* | DQ119839 |
| B | *AP3* | *Dendrobium devonianum* | GU126414 |
| B | *AP3* | *Dendrobium moniliforme* | EU056327 |
| B | *MADS4* | *Dendrobium moniliforme* | GU132995 |
| B | *AP3* | *Dendrobium moniliforme* | EU056328 |
| B | *DEF1* | *Elaeis guineensis* | AY739700 |
| B | *AP3* | *Elegia elephantina* | DQ662239 |
| B | *AP3b* | *Elegia elephantina* | DQ662240 |
| B | *EpMADS13* | *Erycina pusilla* | KJ002738 |
| B | *EpMADS14* | *Erycina pusilla* | KJ002739 |
| B | *EpMADS15* | *Erycina pusilla* | KJ002740 |
| B | *EfAP3* | *Euryale ferox* | AB158349 |
| B | *AP3.1* | *Galeola falconeri* | EU444029 |
| B | *DEF2* | *Gongora galeata* | FJ804098 |
| B | *DEF1* | *Gongora galeata* | FJ804097 |
| B | *DEF3* | *Gongora galeata* | FJ804099 |
| B | *HrDEF* | *Habenaria radiata* | AB232663 |
| B | *AP3* | *Hordeum vulgare* | AY541065 |
| B | *DEF2* | *Hypoxis villosa* | FJ804102 |
| B | *DEF1* | *Hypoxis villosa* | FJ804101 |
| B | *AP3.1* | *Illicium floridanum* | AY936225 |
| B | *AP3.1* | *Illicium henryi* | AY436729 |
| B | *AP3* | *Joinvillea ascendens* | DQ662238 |
| B | *KjAP3* | *Kadsura japonica* | AB154848 |
| B | *AP3* | *Magnolia grandiflora* | AY337752 |
| B | *MaDEF* | *Muscari armeniacum* | AB201751 |
| B | *NjAP3.2* | *Nuphar japonica* | AB158358 |
| B | *AP3.1* | *Nuphar variegata* | AY337745 |
| B | *NtAP3.1* | *Nymphaea tetragona* | AB158351 |
| B | *MADS3* | *Oncidium* Gower-Ramsey | AY196350 |
| B | *MADS5* | *Oncidium* Gower-Ramsey | HM140840 |
| B | *MADS9* | *Oncidium* Gower-Ramsey | HM140841 |
| B | *OitaDEF1* | *Orchis italica* | AB857726 |
| B | *OitaDEF4* | *Orchis italica* | AB857729 |
| B | *OitaDEF3* | *Orchis italica* | AB857728 |
| B | *OitaDEF2* | *Orchis italica* | AB857727 |
| B | *MADS5* | *Phalaenopsis equestris* | AY378148 |
| B | *MADS2* | *Phalaenopsis equestris* | AY378149 |
| B | *MADS4* | *Phalaenopsis equestris* | AY378147 |
| B | *MADS3* | *Phalaenopsis equestris* | AY378150 |
| B | *DEF2* | *Phragmipedium longifolium* | FJ804106 |
| B | *DEF4* | *Phragmipedium longifolium* | FJ804108 |
| B | *DEF3* | *Phragmipedium longifolium* | FJ804107 |
| B | *DEF3* | *Phragmipedium longifolium* | FJ804105 |
| B | *DEF4* | *Spiranthes odorata* | FJ804113 |
| B | *DEF3* | *Spiranthes odorata* | FJ804112 |
| B | *DEF1* | *Spiranthes odorata* | FJ804110 |
| B | *DEF2* | *Spiranthes odorata* | FJ804111 |
| B | *AP3* | *Streptochaeta angustifolia* | DQ662237 |
| B | *AP3* | *Tacca chantieri* | AF230706 |
| B | *TRDEF* | *Tradescantia reflexa* | AB177806 |
| B | *TaMADS#51* | *Triticum aestivum* | AB007506 |
| B | *TaMADS#82* | *Triticum aestivum* | AB107993 |
| B | *DEF1* | *Vanilla planifolia* | FJ804115 |
| B | *DEF2* | *Vanilla planifolia* | FJ804116 |
| B | *DEF3* | *Vanilla planifolia* | FJ804117 |
| B | *silky1* | *Zea mays* | AF181479 |
| B | *CcPI* | *Cabomba caroliniana* | AB158354 |
| B | *PI.1* | *Caryota mitis* | DQ005601 |
| B | *PI.2* | *Caryota mitis* | DQ005600 |
| B | *CCGLO* | *Commelina communis* | AB177807 |
| B | *PI.1* | *Cortaderia selloana* | DQ005583 |
| B | *PI2* | *Cortaderia selloana* | DQ005584 |
| B | *PIC2* | *Crocus sativus* | DQ231251 |
| B | *PI* | *Cymbidium faberi* | HQ164433 |
| B | *GLOBOSA* | *Cymbidium goeringii* | HM106984 |
| B | *PI* | *Dendrobium moniliforme* | EU056326 |
| B | *PI* | *Dendrobium thyrsiflorum* | DQ017701 |
| B | *GLO2* | *Elaeis guineensis* | AF411848 |
| B | *GLO1* | *Elaeis guineensis* | AF227195 |
| B | *PI* | *Elegia elephantina* | DQ662246 |
| B | *PI* | *Epipactis palustris* | DQ005588 |
| B | *EpMADS16* | *Erycina pusilla* | KJ002741 |
| B | *EfPI* | *Euryale ferox* | AB158350 |
| B | *PI* | *Globba marantina* | DQ005602 |
| B | *GLO1* | *Gongora galeata* | FJ804100 |
| B | *PI.2* | *Habenaria petelotii* | EU444035 |
| B | *HrGLO2* | *Habenaria radiata* | AB232664 |
| B | *HrGLO1* | *Habenaria radiata* | AB232665 |
| B | *PI* | *Hordeum vulgare* | AY541066 |
| B | *GLO1* | *Hypoxis villosa* | FJ804103 |
| B | *GLO2* | *Hypoxis villosa* | FJ804104 |
| B | *PI* | *Illicium floridanum* | AY936224 |
| B | *PI.1* | *Illicium henryi* | AY436734 |
| B | *PI* | *Joinvillea ascendens* | DQ662245 |
| B | *KjPI* | *Kadsura japonica* | AB154849 |
| B | *PI.2* | *Ludisia discolor* | EU444040 |
| B | *MADS1* | *Musa acuminata* | AY941798 |
| B | *PIa* | *Musa ornata* | DQ005604 |
| B | *PIb* | *Musa ornata* | DQ005603 |
| B | *PI.2* | *Muscari botryoides* | DQ005596 |
| B | *PI.1* | *Muscari botryoides* | DQ005597 |
| B | *PI.1b* | *Narcissus cyclamineus* | DQ005599 |
| B | *NjPI.1* | *Nuphar japonica* | AB158359 |
| B | *NjPI.2* | *Nuphar japonica* | AB158360 |
| B | *NtPI* | *Nymphaea tetragona* | AB158352 |
| B | *MADS8* | *Oncidium* Gower-Ramsey | HM140842 |
| B | *OrcPI2* | *Orchis italica* | AB537504 |
| B | *OrcPI* | *Orchis italica* | AB094985 |
| B | *MADS4* | *Oryza sativa* | L37527 |
| B | *RMADS219* | *Oryza sativa Japonica Group* | AY551924 |
| B | *GLO* | *Paphiopedilum concolor* | JN565030 |
| B | *MADS6* | *Phalaenopsis equestris* | AY678299 |
| B | *PI2* | *Pharus virescens* | DQ662242 |
| B | *PI1* | *Pharus virescens* | DQ662243 |
| B | *GLO1* | *Phragmipedium longifolium* | FJ804109 |
| B | *PI* | *Sedirea japonica* | KM975642 |
| B | *PI.1a* | *Speirantha convallarioides* | DQ005605 |
| B | *PI.1b* | *Speirantha convallarioides* | DQ005606 |
| B | *GLO1* | *Spiranthes odorata* | FJ804114 |
| B | *PI2* | *Streptochaeta angustifolia* | DQ662241 |
| B | *PI1* | *Streptochaeta angustifolia* | DQ662244 |
| B | *PI* | *Tacca chantieri* | AF230713 |
| B | *WPI1* | *Triticum aestivum* | AB107991 |
| B | *WPI2* | *Triticum aestivum* | AB107992 |
| B | *GLO1* | *Vanilla planifolia* | FJ804118 |
| B | *m18* | *Zea mays* | AJ292960 |
| B | *zmm29* | *Zea mays* | NM001111667 |
| B | *zmm16* | *Zea mays* | NM001111666 |
| C | *AG* | *Allium cepa* | JX974431 |
| C | *MADS6* | *Alpinia hainanensis* | AY621155 |
| C | *AVAG1* | *Asparagus virgatus* | AB125347 |
| C | *AG1* | *Crocus sativus* | AY555579 |
| C | *AG* | *Cycas edentata* | AF492455 |
| C | *AG* | *Cymbidium ensifolium* | JN613149 |
| C | *MADS1* | *Cymbidium ensifolium* | GU123626 |
| C | *MADS2* | *Cymbidium ensifolium* | GU123627 |
| C | *AG1* | *Cymbidium faberi* | JX050151 |
| C | *AG1* | *Dendrobium crumenatum* | DQ119840 |
| C | *DMAG1* | *Dendrobium moniliforme* | EU056329 |
| C | *AG1* | *Dendrobium thyrsiflorum* | DQ017702 |
| C | *AG1* | *Elaeis guineensis* | AY739698 |
| C | *AG2* | *Elaeis guineensis* | AY739699 |
| C | *EpMADS20* | *Erycina pusilla* | KJ002745 |
| C | *EpMADS21* | *Erycina pusilla* | KJ002746 |
| C | *EpMADS22* | *Erycina pusilla* | KJ002747 |
| C | *GBM5* | *Ginkgo biloba* | AY114304 |
| C | *GGM3* | *Gnetum gnemon* | AJ132209 |
| C | *CD28* | *Gongora galeata* | KF914207 |
| C | *AG* | *Hosta plantaginea* | EU429307 |
| C | *HAG1* | *Hyacinthus orientalis* | AF099937 |
| C | *2A* | *Hypoxis villosa* | KF914210 |
| C | *AG1* | *Lilium formosanum* | HQ234917 |
| C | *LFMADS1* | *Lilium longiflorum* | AY829227 |
| C | *AG1* | *Lilium longiflorum* | AY500376 |
| C | *MADS* | *Narcissus tazetta* | EF421828 |
| C | *MADS4* | *Oncidium* Gower-Ramsey | KJ819939 |
| C | *AG* | *Orchis italica* | JX205496 |
| C | *KCS268A08* | *Oryza sativa* | FJ750942 |
| C | *MADS3* | *Oryza sativa* | L37528 |
| C | *MADS1* | *Phalaenopsis equestris* | AF234617 |
| C | *MADS.box* | *Phragmipedium longifolium* | KF914208 |
| C | *dal2* | *Picea abies* | X79280 |
| C | *MADS.box* | *Vanilla planifolia* | KF914209 |
| C | *ZAG1* | *Zea mays* | NM001111851 |
| C | *AG* | *Allium cepa* | JX974431 |
| C | *MADS6* | *Alpinia hainanensis* | AY621155 |
| C | *AVAG1* | *Asparagus virgatus* | AB125347 |
| C | *AG1* | *Crocus sativus* | AY555579 |
| C | *AG* | *Cycas edentata* | AF492455 |
| C | *AG* | *Cymbidium ensifolium* | JN613149 |
| C | *MADS1* | *Cymbidium ensifolium* | GU123626 |
| C | *MADS2* | *Cymbidium ensifolium* | GU123627 |
| C | *AG1* | *Cymbidium faberi* | JX050151 |
| C | *AG1* | *Dendrobium crumenatum* | DQ119840 |
| C | *DMAG1* | *Dendrobium moniliforme* | EU056329 |
| C | *AG1* | *Dendrobium thyrsiflorum* | DQ017702 |
| C | *AG1* | *Elaeis guineensis* | AY739698 |
| C | *AG2* | *Elaeis guineensis* | AY739699 |
| C | *AG* | *Erycina pusilla* | KJ002745 |
| C | *AG* | *Erycina pusilla* | KJ002746 |
| C | *AG* | *Erycina pusilla* | KJ002747 |
| C | *GBM5* | *Ginkgo biloba* | AY114304 |
| C | *GGM3* | *Gnetum gnemon* | AJ132209 |
| C | *CD28* | *Gongora galeata* | KF914207 |
| C | *AG* | *Hosta plantaginea* | EU429307 |
| C | *HAG1* | *Hyacinthus orientalis* | AF099937 |
| C | *2A* | *Hypoxis villosa* | KF914210 |
| C | *AG1* | *Lilium formosanum* | HQ234917 |
| C | *LFMADS1* | *Lilium longiflorum* | AY829227 |
| C | *AG1* | *Lilium longiflorum* | AY500376 |
| C | *MADS* | *Narcissus tazetta* | EF421828 |
| C | *MADS4* | *Oncidium* Gower-Ramsey | KJ819939 |
| C | *AG* | *Orchis italica* | JX205496 |
| C | *KCS268A08* | *Oryza sativa* | FJ750942 |
| C | *MADS3* | *Oryza sativa* | L37528 |
| C | *MADS1* | *Phalaenopsis equestris* | AF234617 |
| C | *MADS.box* | *Phragmipedium longifolium* | KF914208 |
| C | *dal2* | *Picea abies* | X79280 |
| C | *MADS.box* | *Vanilla planifolia* | KF914209 |
| C | *ZAG1* | *Zea mays* | NM001111851 |
| D | *ApMADS2* | *Agapanthus praecox* | AB079260 |
| D | *AVAG2* | *Asparagus virgatus* | AB175825 |
| D | *AG-C* | *Cycas edentata* | AF492455 |
| D | *MADS3* | *Cymbidium ensifolium* | JN613150 |
| D | *AG2* | *Dendrobium crumenatum* | DQ119841 |
| D | *MADS2* | *Dendrobium nobile* | EF535599 |
| D | *AG2* | *Dendrobium thyrsiflorum* | DQ017703 |
| D | *EpMADS23* | *Erycina pusilla* | KJ002748 |
| D | *GBM5-C* | *Ginkgo biloba* | AY114304 |
| D | *GGM3-C* | *Gnetum gnemon* | AJ132209 |
| D | *MADS.box* | *Gongora galeata* | KF914206 |
| D | *MADS1* | *Hyacinthus orientalis* | AF194335 |
| D | *MADS.box* | *Hypoxis villosa* | KF914205 |
| D | *STK* | *Lacandonia schismatica* | GQ214164 |
| D | *MADS2* | *Lilium longiflorum* | AY522502 |
| D | *MADS5* | *Musa acuminata* | EU869310 |
| D | *MADS2* | *Oncidium* Gower-Ramsey | KJ819938 |
| D | *STK* | *Orchis italica* | JX205497 |
| D | *MADS13* | *Oryza sativa* | AF151693 |
| D | *MADS21* | *Oryza sativa* | AY177693 |
| D | *MADS7* | *Phalaenopsis equestris* | JN983500 |
| D | *dal2-C* | *Picea abies* | X79280 |
| D | *m25* | *Zea mays* | AJ430639 |
| D | *zag2* | *Zea mays* | NM001111908 |
| D | *ov23* | *Zea mays* | NM001111909 |
| E | *AGL2* | *Acorus americanus* | AY850184 |
| E | *AlsSEPa* | *Alstroemeria ligtu* | AB694892 |
| E | *AlsSEPd* | *Alstroemeria ligtu* | AB694895 |
| E | *AlsSEPb* | *Alstroemeria ligtu* | AB694893 |
| E | *AGL2* | *Amborella trichopoda* | AY850179 |
| E | *SEP* | *Aranda deborah* | X69107 |
| E | *MADS600* | *Asarum caudigerum* | AJ419956 |
| E | *MADS2* | *Asparagus officinalis* | DQ344503 |
| E | *AOM1* | *Asparagus officinalis* | AY382400 |
| E | *CraOM1* | *Cleisostoma racemiferum* | AY056827 |
| E | *SEP3b* | *Crocus sativus* | EU424138 |
| E | *SEP3a* | *Crocus sativus* | EU424137 |
| E | *SEP3c* | *Crocus sativus* | EU424139 |
| E | *SEP* | *Cymbidium ensifolium* | JQ326258 |
| E | *SEP3* | *Cymbidium goeringii* | KF924272 |
| E | *DcOSEP1* | *Dendrobium crumenatum* | DQ119842 |
| E | *SEP3* | *Dendrobium nobile* | HQ388352 |
| E | *EgAGL2.3* | *Elaeis guineensis* | AF411845 |
| E | *EgAGL2.2* | *Elaeis guineensis* | AF411844 |
| E | *EgAGL2.1* | *Elaeis guineensis* | AF411843 |
| E | *mads8* | *Elaeis guineensis* | AJ581461 |
| E | *EgAGL2.4* | *Elaeis guineensis* | AF411846 |
| E | *EpMADS6* | *Erycina pusilla* | KJ002731 |
| E | *EpMADS7* | *Erycina pusilla* | KJ002732 |
| E | *EpMADS8* | *Erycina pusilla* | KJ002733 |
| E | *EpMADS9* | *Erycina pusilla* | KJ002734 |
| E | *MADS.box* | *Gongora galeata* | KF914200 |
| E | *HcAP1* | *Houttuynia cordata* | AB089153 |
| E | *HcSEP2* | *Houttuynia cordata* | AB089158 |
| E | *HcSEP3* | *Houttuynia cordata* | AB089159 |
| E | *E.3S3* | *Hypoxis villosa* | KF914203 |
| E | *E.1S* | *Hypoxis villosa* | KF914204 |
| E | *AGL9* | *Liriodendron tulipifera* | AY850182 |
| E | *LITF.MADS.9* | *Lycoris longituba* | GQ166037 |
| E | *LITFMADS15* | *Lycoris longituba* | GQ166148 |
| E | *AGL9* | *Magnolia grandiflora* | AY821782 |
| E | *MpMADS13* | *Magnolia praecocissima* | AB050655 |
| E | *MADS2* | *Musa acuminata* | EU869306 |
| E | *MADS4* | *Musa acuminata* | EU869309 |
| E | *MADS6* | *Oncidium* Gower-Ramsey | HM140844 |
| E | *MADS11* | *Oncidium* Gower-Ramsey | HM140847 |
| E | *MADS7* | *Oryza sativa* | U78891 |
| E | *MADS1* | *Oryza sativa* | AY895163 |
| E | *SEP1* | *Paphiopedilum concolor* | JQ030893 |
| E | *AGL9.1* | *Persea americana* | AY850185 |
| E | *AGL9.2* | *Persea americana* | AY850186 |
| E | *PeSEP1* | *Phalaenopsis equestris* | KF673857 |
| E | *PeSEP2* | *Phalaenopsis equestris* | KF673858 |
| E | *PeSEP3* | *Phalaenopsis equestris* | KF673859 |
| E | *PeSEP4* | *Phalaenopsis equestris* | KF673860 |
| E | *MADS.box* | *Vanilla planifolia* | KF914202 |
| E | *ZMM3* | *Zea mays* | Y09301 |
| E | *ZMM7* | *Zea mays* | Y09302 |
| E | *zmm27* | *Zea mays* | NM001112055 |
| E | *ZMM8* | *Zea mays* | Y09303 |
| E | *ZmM6* | *Zea mays* | NM001111683 |
| E | *zmm24* | *Zea mays* | NM001111679 |
| E | *zmm31* | *Zea mays* | NM001111680 |
| A/E | *AlsAGL6* | *Alstroemeria ligtu* | AB694896 |
| A/E | *AncomAGL6* | *Ananas comosus* | KC257408 |
| A/E | *AoAGL6* | *Asparagus officinalis* | AY383559 |
| A/E | *AGL6* | *Bambusa oldhamii* | EF517293 |
| A/E | *AGL6* | *Brachypodium distachyon* | NM001302881 |
| A/E | *AGL6* | *Cymbidium ensifolium* | JN613148 |
| A/E | *AGL6* | *Cymbidium faberi* | HM208534 |
| A/E | *AGL6* | *Cymbidium goeringii* | GQ265900 |
| A/E | *AGL6* | *Cymbidium goeringii* | HM208533 |
| A/E | *EgAGL6* | *Elaeis guineensis* | AY739701 |
| A/E | *EpMADS3* | *Erycina pusilla* | KJ002728 |
| A/E | *EpMADS4* | *Erycina pusilla* | KJ002729 |
| A/E | *EpMADS5* | *Erycina pusilla* | KJ002730 |
| A/E | *GbMADS8* | *Ginkgo biloba* | AB029470 |
| A/E | *GbMADS1* | *Ginkgo biloba* | AB029463 |
| A/E | *ggm11* | *Gnetum gnemon* | AJ132217 |
| A/E | *ggm9* | *Gnetum gnemon* | AJ132215 |
| A/E | *HvAGL6* | *Hordeum vulgare* | AY541067 |
| A/E | *JaAGL6* | *Joinvillea ascendens* | GQ496631 |
| A/E | *NtAGL6A* | *Narcissus tazetta* | EU081900 |
| A/E | *NtAGL6B* | *Narcissus tazetta* | EF517294 |
| A/E | *AGL6* | *Oncidium* Gower-Ramsey | HM140845 |
| A/E | *MADS1* | *Oncidium* Gower-Ramsey | HM140843 |
| A/E | *MADS6* | *Oryza sativa* | FJ750939 |
| A/E | *MADS17* | *Oryza sativa* | FJ668596 |
| A/E | *MADS6* | *Phyllostachys edulis* | KJ002715 |
| A/E | *PeAGL6* | *Phyllostachys edulis* | EU327784 |
| A/E | *DAL14* | *Picea abies* | KC347012 |
| A/E | *PaDAL1* | *Picea abies* | X80902 |
| A/E | *PrMADS2* | *Pinus radiata* | U42400 |
| A/E | *PrMADS3* | *Pinus radiata* | U76726 |
| A/E | *SiAGL6A* | *Setaria italica* | GQ496658 |
| A/E | *ZAG5* | *Zea mays* | NM001112222 |

**Table S2:** Transcript primer sequences and amplicon characteristics used for quantitative real-time PCR validation of the expression profiles of eighteen MADS box transcripts, following MIQE guidelines [(Bustin *et al.* 200](#h.30j0zll)[9](#_ENREF_1)). All sequences listed here listed were either downloaded from NCBI GenBank or from the Orchidstra database http://orchidstra.abrc.sinica.edu.tw.

| **EST No.**  **Orchidstra** | **Accession** | **Target**  **Gene** | **Class** | **MADS** | **Primer**  **Name** | **Sequence** | **Tm (°C)** | **GC (%)** | **Amplicon (bp)** |
| --- | --- | --- | --- | --- | --- | --- | --- | --- | --- |
| EPTC002102 |  | Actin |  |  | EpAct-fw1 BD | AGTATAGTGTCTGGATTGGAGGAT | 64,1 | 41,6 | 142 |
|  |  |  |  |  | EpAct-rv1 BD | GGAAGGCAGTAATAATGGACTCAG | 64,9 | 45,8 |  |
| EPTC010048 |  | UBI2 |  |  | EpUbi2-2fw | CGTTGATGAGTTCTGAGGATGATT | 65,2 | 41,6 | 133 |
|  |  |  |  |  | EpUbi2-2rv | CAAGCAAGTTGACAAATAAAGTAGCA | 64,1 | 34,6 |  |
| EPTC011841 |  | Fbox |  |  | EpFbox-fw1 | TACCTCAGCATATAACGGACCAT | 63,2 | 43,4 | 90 |
|  |  |  |  |  | EpFbox-rv2 | CGCACATAGCCGCAATTCTT | 66,1 | 52,6 |  |
|  | KJ002741 | PI | B | 16 | EpPI fw546 | GGCAATGGAAGGCAGCATGAGA | 72,4 | 54,5 | 110 |
|  |  |  |  |  | EpPI rv655 | TGGGTTGAATGGGTTGCACTCGA | 74,4 | 52,1 |  |
|  | KJ002738 | AP3 | B | 13 | EpAP3A1 fw2 | GGAATCGGAAGTATCATGTCTATCAG | 66,4 | 44,0 | 82 |
|  |  |  |  |  | EpAP3A1 rv2 | TTTCTGTGGGTTTCTTGAGAGTTC | 61,4 | 41,6 |  |
|  | KJ002740 | AP3 | B | 15 | EpAP3B1 fw2 | AATTCCAATGGCTACTGAATGTCC | 65,8 | 41,6 | 93 |
|  |  |  |  |  | EpAP3B1 rv2 | TGAGATTCATAACCCAAACCAAGT | 64,3 | 37,5 |  |
|  | KJ002739 | AP3 | B | 14 | EpAP3B2 fw2 | GCTACGCAAACGGACACTT | 62,9 | 52,6 | 83 |
|  |  |  |  |  | EpAP3B2 rv2 | ACCTCTTGCAGCTCATTCAATAG | 63,4 | 43,4 |  |
|  | KJ002745 | AG | C | 20 | MADS20 Fw2 | AGAGTGAGACAGCAGCAGAT | 64,9 | 50,0 | 136 |
|  |  |  |  |  | MADS20 Rv2 | AGCTTAATTAGTTGGATGGTAGGC | 64,6 | 41,7 |  |
|  | KJ002746 | AG | C | 21 | MADS21 Fw1 | GCCTTCAACAAGCACAGATTATG | 64,5 | 43,4 | 140 |
|  |  |  |  |  | MADS21 Rv1 | GCAACAATGTCCCTTACCCAA | 65,7 | 47,6 |  |
|  | KJ002747 | AG | C | 22 | MADS22 Fw1 | CCCAACCATCATTATTCTCTCCAA | 64,6 | 41,7 | 124 |
|  |  |  |  |  | MADS22 Rv1 | TCTGCCAAACAAATTCACTCCA | 64,9 | 40,9 |  |
|  | KJ002748 | STK | D | 23 | MADS23 Fw1 | AGACAAATATGGTACAAGCAGGAG | 64,6 | 41,7 | 112 |
|  |  |  |  |  | MADS23 Rv1 | TGATGGTGTGAATAAGGAGGTG | 64,0 | 45,5 |  |
|  | KJ002735 | FUL | A | 10 | EpMADS10 Fw1 | CATCCACGCCACCAACTG | 65,3 | 61,1 | 84 |
|  |  |  |  |  | EpMADS10 Rv1 | AGACACCTGAGGGACACTTT | 64,8 | 50,0 |  |
|  | KJ002736 | FUL | A | 11 | EpMADS11 Fw2 | TCTCCTCGCCTGCTTCTTAC | 65,5 | 55,0 | 96 |
|  |  |  |  |  | EpMADS11 Rv2 | GCTGTGCTATCTCCTCACCAT | 65,9 | 52,4 |  |
|  | KJ002737 | FUL | A | 12 | EpMADS12 Fw1 | GCTTGTAGAAGGCTTGTTGC | 63,9 | 50,0 | 92 |
|  |  |  |  |  | EpMADS12 Rv1 | AGTGCCGCTAATGGAAATATAGAA | 64,1 | 37,5 |  |
|  | KJ002728 | AGL6 | A/E | 3 | EpMADS3 Fw1 | GAATCTGATGCTGTAGTTGATGGA | 64,6 | 41,7 | 89 |
|  |  |  |  |  | EpMADS3 Rv1 | CCTATATGGAGAGTCGGTTCACA | 65,2 | 47,8 |  |
|  | KJ002729 | AGL6 | A/E | 4 | EpMADS4 Fw2 | TCGCTATGAAGCATCTGAAGAAC | 64,9 | 43,5 | 83 |
|  |  |  |  |  | EpMADS4 Rv1 | TTTATCAAAGAGTCCAACCCTGTG | 64,9 | 41,7 |  |
|  | KJ002730 | AGL6 | A/E | 5 | EpMADS5 Fw1 | AAGAGCAATTCAAGGTTCCAACT | 64,9 | 39,1 | 99 |
|  |  |  |  |  | EpMADS5 Rv1 | TGAAGAGTAGGTTCTGTGTCCAT | 65,0 | 43,5 |  |
|  | KJ002731 | SEP | E | 6 | EpMADS6 Fw2 | CGTCAGCAGCCTCAATCAG | 65,0 | 57,9 | 117 |
|  |  |  |  |  | EpMADS6 Rv1 | ATGACCCTGTGTTCAACTGTT | 64,1 | 42,9 |  |
|  | KJ002732 | SEP | E | 7 | EpMADS7 Fw1 | CGTCAGCCTCAATCAGATGTT | 64,6 | 47,6 | 100 |
|  |  |  |  |  | EpMADS7 Rv1 | CGTTGTTCAAATGTTGTTCTATGC | 63,6 | 37,5 |  |
|  | KJ002733 | SEP | E | 8 | EpMADS8 Fw1 | TTGGAGTGCGAGCCTACA | 64,9 | 55,6 | 96 |
|  |  |  |  |  | EpMADS8 Rv2 | TCCTGGTGGCATGTAATTGTG | 64,8 | 47,6 |  |
|  | KJ002734 | SEP | E | 9 | EpMADS9 Fw1 | CAGCTCAGCACCATGAAGAT | 64,3 | 50,0 | 141 |
|  |  |  |  |  | EpMADS9 Rv1 | GTCCAAGCCAGCCAGATG | 64,7 | 61,1 |  |

**Table S3**: Difference in MADS-box gene expression between flower organs; variance analysis of measures using Tukey multicomparisons test.

| **Class A** | **Floral** | **Floral** |  | **Class A** | **Floral** | **Floral** |  | **Class A** | **Floral** | **Floral** |
| --- | --- | --- | --- | --- | --- | --- | --- | --- | --- | --- |
| **EpMADS10** | **Stage 2** | **Stage 4** |  | **EpMADS11** | **Stage 2** | **Stage 4** |  | **EpMADS12** | **Stage 2** | **Stage 4** |
| lse vs. mse | *** | ns |  | lse vs. mse | ns | ns |  | lse vs. mse | ns | ns |
| lse vs. lip | **** | ns |  | lse vs. lip | ns | ns |  | lse vs. lip | ns | ns |
| lse vs. cl | **** | ns |  | lse vs. cl | ns | ns |  | lse vs. cl | ns | ns |
| lse vs. pe | ns | ns |  | lse vs. pe | ns | ns |  | lse vs. pe | ns | ns |
| lse vs. fs | *** | ns |  | lse vs. fs | ns | ns |  | lse vs. fs | ns | ns |
| lse vs. gm | ** | ns |  | lse vs. gm | ns | ns |  | lse vs. gm | ns | ns |
| mse vs. lip | ns | ns |  | mse vs. lip | ns | ns |  | mse vs. lip | ns | ns |
| mse vs. cl | ** | ns |  | mse vs. cl | ns | ns |  | mse vs. cl | ns | ns |
| mse vs. pe | **** | ns |  | mse vs. pe | ns | ns |  | mse vs. pe | ns | ns |
| mse vs. fs | ns | ns |  | mse vs. fs | ns | ns |  | mse vs. fs | ns | ns |
| mse vs. gm | ns | ns |  | mse vs. gm | ns | ns |  | mse vs. gm | ns | ns |
| lip vs. cl | ns | ns |  | lip vs. cl | ns | ns |  | lip vs. cl | ns | ns |
| lip vs. pe | **** | ns |  | lip vs. pe | ns | ns |  | lip vs. pe | ns | ns |
| lip vs. fs | ns | ns |  | lip vs. fs | ns | ns |  | lip vs. fs | ns | ns |
| lip vs. gm | ns | ns |  | lip vs. gm | ns | ns |  | lip vs. gm | ns | ns |
| cl vs. pe | **** | ** |  | cl vs. pe | ns | ns |  | cl vs. pe | ns | ns |
| cl vs. fs | ** | ns |  | cl vs. fs | ns | ns |  | cl vs. fs | ns | ns |
| cl vs. gm | *** | ns |  | cl vs. gm | ns | ns |  | cl vs. gm | ns | ns |
| pe vs. fs | **** | ns |  | pe vs. fs | ns | ns |  | pe vs. fs | ns | ns |
| pe vs. gm | **** | ns |  | pe vs. gm | ns | * |  | pe vs. gm | ns | ns |
| fs vs. gm | ns | ns |  | fs vs. gm | ns | ns |  | fs vs. gm | ns | ns |

P-value style: GP: >0.05 (ns), <0.05 (*), <0.01 (**), <0.001 (***), <0.0001 (****). Abbreviations: lse = lateral sepal, mse = median sepal, cl = callus, pe = petal, fs = fertile stamen and gm = gynostemium.

| **Class B** | **Floral** | **Floral** |  | **Class B** | **Floral** | **Floral** |  | **Class B** | **Floral** | **Floral** |  | **Class B** | **Floral** | **Floral** |
| --- | --- | --- | --- | --- | --- | --- | --- | --- | --- | --- | --- | --- | --- | --- |
| **EpMADS13** | **Stage 2** | **Stage 4** |  | **EpMADS14** | **Stage 2** | **Stage 4** |  | **EpMADS15** | **Stage 2** | **Stage 4** |  | **EpMADS16** | **Stage 2** | **Stage 4** |
| lse vs. mse | ns | ns |  | lse vs. mse | **** | **** |  | lse vs. mse | *** | ns |  | lse vs. mse | ns | ns |
| lse vs. lip | ns | ns |  | lse vs. lip | **** | **** |  | lse vs. lip | ns | **** |  | lse vs. lip | ns | ns |
| lse vs. cl | **** | * |  | lse vs. cl | **** | **** |  | lse vs. cl | ns | **** |  | lse vs. cl | ns | ns |
| lse vs. pe | *** | **** |  | lse vs. pe | **** | **** |  | lse vs. pe | ns | ns |  | lse vs. pe | ns | ns |
| lse vs. fs | ns | ns |  | lse vs. fs | **** | **** |  | lse vs. fs | ns | ns |  | lse vs. fs | *** | ** |
| lse vs. gm | * | ns |  | lse vs. gm | **** | **** |  | lse vs. gm | ** | ns |  | lse vs. gm | * | ns |
| mse vs. lip | ns | ns |  | mse vs. lip | * | **** |  | mse vs. lip | **** | **** |  | mse vs. lip | ns | ns |
| mse vs. cl | **** | ns |  | mse vs. cl | ns | **** |  | mse vs. cl | **** | **** |  | mse vs. cl | ns | ns |
| mse vs. pe | *** | ** |  | mse vs. pe | ns | ns |  | mse vs. pe | ns | ns |  | mse vs. pe | ns | ns |
| mse vs. fs | ns | ns |  | mse vs. fs | ns | *** |  | mse vs. fs | **** | ns |  | mse vs. fs | ** | **** |
| mse vs. gm | ns | ns |  | mse vs. gm | ns | ** |  | mse vs. gm | ns | ns |  | mse vs. gm | ns | ** |
| lip vs. cl | ** | ns |  | lip vs. cl | ns | ns |  | lip vs. cl | ns | ns |  | lip vs. cl | ns | ns |
| lip vs. pe | ns | *** |  | lip vs. pe | ns | ** |  | lip vs. pe | **** | **** |  | lip vs. pe | ns | * |
| lip vs. fs | ns | ns |  | lip vs. fs | ns | ns |  | lip vs. fs | ns | **** |  | lip vs. fs | * | * |
| lip vs. gm | ns | ns |  | lip vs. gm | ns | ns |  | lip vs. gm | **** | **** |  | lip vs. gm | ns | ns |
| cl vs. pe | ns | ns |  | cl vs. pe | ns | *** |  | cl vs. pe | *** | *** |  | cl vs. pe | * | ns |
| cl vs. fs | **** | ns |  | cl vs. fs | ns | ns |  | cl vs. fs | ns | **** |  | cl vs. fs | ns | ** |
| cl vs. gm | * | ns |  | cl vs. gm | ns | ns |  | cl vs. gm | **** | **** |  | cl vs. gm | ns | ns |
| pe vs. fs | ** | *** |  | pe vs. fs | ns | * |  | pe vs. fs | * | ns |  | pe vs. fs | **** | **** |
| pe vs. gm | ns | ns |  | pe vs. gm | ns | ns |  | pe vs. gm | ns | ** |  | pe vs. gm | ** | *** |
| fs vs. gm | ns | ns |  | fs vs. gm | ns | ns |  | fs vs. gm | ** | ns |  | fs vs. gm | ns | ns |

P-value style: GP: >0.05 (ns), <0.05 (*), <0.01 (**), <0.001 (***), <0.0001 (****). Abbreviations: lse = lateral sepal, mse = median sepal, cl = callus, pe = petal, fs = fertile stamen and gm = gynostemium.

| **Class C** | **Floral** | **Floral** |  | **Class C** | **Floral** | **Floral** |  | **Class C** | **Floral** | **Floral** |  | **Class D** | **Floral** | **Floral** |
| --- | --- | --- | --- | --- | --- | --- | --- | --- | --- | --- | --- | --- | --- | --- |
| **EpMADS20** | **Stage 2** | **Stage 4** |  | **EpMADS21** | **Stage 2** | **Stage 4** |  | **EpMADS22** | **Stage 2** | **Stage 4** |  | **EpMADS23** | **Stage 2** | **Stage 4** |
| lse vs. mse | ns | ns |  | lse vs. mse | ns | ns |  | lse vs. mse | ns | ns |  | lse vs. mse | ns | ns |
| lse vs. lip | ns | ns |  | lse vs. lip | ns | ns |  | lse vs. lip | ns | ns |  | lse vs. lip | ns | ns |
| lse vs. cl | ns | ns |  | lse vs. cl | ns | ns |  | lse vs. cl | ns | ns |  | lse vs. cl | ns | ns |
| lse vs. pe | ns | ns |  | lse vs. pe | ns | ns |  | lse vs. pe | ns | ns |  | lse vs. pe | ns | ns |
| lse vs. fs | ns | ns |  | lse vs. fs | ns | ns |  | lse vs. fs | ns | **** |  | lse vs. fs | ns | ns |
| lse vs. gm | **** | * |  | lse vs. gm | ns | **** |  | lse vs. gm | * | ** |  | lse vs. gm | **** | ** |
| mse vs. lip | ns | *** |  | mse vs. lip | ns | ns |  | mse vs. lip | ns | ns |  | mse vs. lip | ns | ns |
| mse vs. cl | ns | *** |  | mse vs. cl | ns | ns |  | mse vs. cl | ns | ns |  | mse vs. cl | ns | ns |
| mse vs. pe | ns | ns |  | mse vs. pe | ns | ns |  | mse vs. pe | ns | ns |  | mse vs. pe | ns | ns |
| mse vs. fs | ns | * |  | mse vs. fs | ns | ns |  | mse vs. fs | ns | **** |  | mse vs. fs | ns | ns |
| mse vs. gm | **** | ns |  | mse vs. gm | * | **** |  | mse vs. gm | * | ** |  | mse vs. gm | **** | ** |
| lip vs. cl | ns | ns |  | lip vs. cl | ns | ns |  | lip vs. cl | ns | ns |  | lip vs. cl | ns | ns |
| lip vs. pe | ns | ** |  | lip vs. pe | ns | ns |  | lip vs. pe | ns | ns |  | lip vs. pe | ns | ns |
| lip vs. fs | ns | ns |  | lip vs. fs | ns | ns |  | lip vs. fs | ns | **** |  | lip vs. fs | ns | ns |
| lip vs. gm | **** | **** |  | lip vs. gm | * | **** |  | lip vs. gm | ** | ** |  | lip vs. gm | **** | * |
| cl vs. pe | ns | ** |  | cl vs. pe | ns | ns |  | cl vs. pe | ns | ns |  | cl vs. pe | ns | ns |
| cl vs. fs | ns | ns |  | cl vs. fs | ns | ns |  | cl vs. fs | ns | **** |  | cl vs. fs | ns | ns |
| cl vs. gm | **** | **** |  | cl vs. gm | * | **** |  | cl vs. gm | * | ** |  | cl vs. gm | **** | ** |
| pe vs. fs | ns | ns |  | pe vs. fs | ns | ns |  | pe vs. fs | ns | **** |  | pe vs. fs | ns | ns |
| pe vs. gm | **** | ns |  | pe vs. gm | * | **** |  | pe vs. gm | ns | ** |  | pe vs. gm | **** | ** |
| fs vs. gm | **** | ** |  | fs vs. gm | ns | * |  | fs vs. gm | ns | ns |  | fs vs. gm | **** | ** |

P-value style: GP: >0.05 (ns), <0.05 (*), <0.01 (**), <0.001 (***), <0.0001 (****). Abbreviations: lse = lateral sepal, mse = median sepal, cl = callus, pe = petal, fs = fertile stamen and gm = gynostemium.

| **Class E** | **Floral** | **Floral** |  | **Class E** | **Floral** | **Floral** |  | **Class E** | **Floral** | **Floral** |  | **Class E** | **Floral** | **Floral** |
| --- | --- | --- | --- | --- | --- | --- | --- | --- | --- | --- | --- | --- | --- | --- |
| **EpMADS6** | **Stage 2** | **Stage 4** |  | **EpMADS7** | **Stage 2** | **Stage 4** |  | **EpMADS8** | **Stage 2** | **Stage 4** |  | **EpMADS9** | **Stage 2** | **Stage 4** |
| lse vs. mse | ns | ns |  | lse vs. mse | ns | ns |  | lse vs. mse | ns | ns |  | lse vs. mse | ** | ns |
| lse vs. lip | ns | ns |  | lse vs. lip | ns | ** |  | lse vs. lip | ns | ns |  | lse vs. lip | ns | **** |
| lse vs. cl | ns | ns |  | lse vs. cl | ns | ns |  | lse vs. cl | ns | ns |  | lse vs. cl | **** | **** |
| lse vs. pe | ns | ns |  | lse vs. pe | ns | ns |  | lse vs. pe | ns | ns |  | lse vs. pe | ns | ns |
| lse vs. fs | *** | **** |  | lse vs. fs | ns | ns |  | lse vs. fs | ** | ns |  | lse vs. fs | ns | **** |
| lse vs. gm | ns | ns |  | lse vs. gm | ns | ns |  | lse vs. gm | ns | ns |  | lse vs. gm | ns | ** |
| mse vs. lip | ns | ns |  | mse vs. lip | ns | ns |  | mse vs. lip | * | ns |  | mse vs. lip | * | **** |
| mse vs. cl | ns | ns |  | mse vs. cl | ns | ns |  | mse vs. cl | ** | ns |  | mse vs. cl | ns | **** |
| mse vs. pe | ns | ns |  | mse vs. pe | * | ns |  | mse vs. pe | ns | ns |  | mse vs. pe | *** | ns |
| mse vs. fs | *** | **** |  | mse vs. fs | ns | ns |  | mse vs. fs | *** | ns |  | mse vs. fs | **** | **** |
| mse vs. gm | ns | ns |  | mse vs. gm | ns | ns |  | mse vs. gm | ** | ns |  | mse vs. gm | ** | ** |
| lip vs. cl | ns | ns |  | lip vs. cl | ns | ns |  | lip vs. cl | ns | ns |  | lip vs. cl | *** | ns |
| lip vs. pe | ns | ns |  | lip vs. pe | ns | ns |  | lip vs. pe | ns | ns |  | lip vs. pe | ns | **** |
| lip vs. fs | **** | **** |  | lip vs. fs | ns | ns |  | lip vs. fs | ns | ns |  | lip vs. fs | ns | ns |
| lip vs. gm | ns | ns |  | lip vs. gm | ns | ** |  | lip vs. gm | ns | ns |  | lip vs. gm | ns | ns |
| cl vs. pe | ns | ns |  | cl vs. pe | ns | ns |  | cl vs. pe | * | ns |  | cl vs. pe | **** | **** |
| cl vs. fs | **** | **** |  | cl vs. fs | ns | ns |  | cl vs. fs | ns | ns |  | cl vs. fs | **** | ns |
| cl vs. gm | ns | ns |  | cl vs. gm | ns | ns |  | cl vs. gm | ns | ns |  | cl vs. gm | **** | * |
| pe vs. fs | *** | **** |  | pe vs. fs | ns | ns |  | pe vs. fs | ** | ns |  | pe vs. fs | ns | **** |
| pe vs. gm | ns | ns |  | pe vs. gm | ns | ns |  | pe vs. gm | * | ns |  | pe vs. gm | ns | ** |
| fs vs. gm | ** | **** |  | fs vs. gm | ns | ns |  | fs vs. gm | ns | ns |  | fs vs. gm | ns | * |

P-value style: GP: >0.05 (ns), <0.05 (*), <0.01 (**), <0.001 (***), <0.0001 (****). Abbreviations: lse = lateral sepal, mse = median sepal, cl = callus, pe = petal, fs = fertile stamen and gm = gynostemium.

| **AGL6** | **Floral** | **Floral** |  | **AGL6** | **Floral** | **Floral** |  | **AGL6** | **Floral** | **Floral** |
| --- | --- | --- | --- | --- | --- | --- | --- | --- | --- | --- |
| **EpMADS3** | **Stage 2** | **Stage 4** |  | **EpMADS4** | **Stage 2** | **Stage 4** |  | **EpMADS5** | **Stage 2** | **Stage 4** |
| lse vs. mse | **** | **** |  | lse vs. mse | **** | ns |  | lse vs. mse | ns | ns |
| lse vs. lip | **** | **** |  | lse vs. lip | **** | ns |  | lse vs. lip | **** | ns |
| lse vs. cl | **** | **** |  | lse vs. cl | **** | ns |  | lse vs. cl | **** | **** |
| lse vs. pe | **** | **** |  | lse vs. pe | ** | ns |  | lse vs. pe | ns | ns |
| lse vs. fs | **** | **** |  | lse vs. fs | **** | ns |  | lse vs. fs | ns | ns |
| lse vs. gm | **** | **** |  | lse vs. gm | ** | ns |  | lse vs. gm | ns | ns |
| mse vs. lip | **** | **** |  | mse vs. lip | ns | ns |  | mse vs. lip | **** | ns |
| mse vs. cl | **** | **** |  | mse vs. cl | ns | ns |  | mse vs. cl | **** | **** |
| mse vs. pe | * | *** |  | mse vs. pe | ns | ns |  | mse vs. pe | ns | ns |
| mse vs. fs | **** | **** |  | mse vs. fs | ns | ns |  | mse vs. fs | ns | ns |
| mse vs. gm | **** | **** |  | mse vs. gm | ns | *** |  | mse vs. gm | ns | ns |
| lip vs. cl | ns | ns |  | lip vs. cl | ns | ns |  | lip vs. cl | ns | **** |
| lip vs. pe | **** | *** |  | lip vs. pe | ns | ns |  | lip vs. pe | **** | ns |
| lip vs. fs | ns | ns |  | lip vs. fs | ns | ns |  | lip vs. fs | **** | ns |
| lip vs. gm | ns | ns |  | lip vs. gm | ns | *** |  | lip vs. gm | **** | ns |
| cl vs. pe | **** | * |  | cl vs. pe | ns | ns |  | cl vs. pe | **** | **** |
| cl vs. fs | ns | ns |  | cl vs. fs | ns | ns |  | cl vs. fs | **** | **** |
| cl vs. gm | ns | ns |  | cl vs. gm | ns | ** |  | cl vs. gm | **** | **** |
| pe vs. fs | **** | *** |  | pe vs. fs | ns | ns |  | pe vs. fs | ns | ns |
| pe vs. gm | **** | *** |  | pe vs. gm | ns | * |  | pe vs. gm | ns | ns |
| fs vs. gm | ns | ns |  | fs vs. gm | ns | *** |  | fs vs. gm | ns | ns |

P-value style: GP: >0.05 (ns), <0.05 (*), <0.01 (**), <0.001 (***), <0.0001 (****). Abbreviations: lse = lateral sepal, mse = median sepal, cl = callus, pe = petal, fs = fertile stamen and gm = gynostemium.
