# Supplementary material for: Exploring the evolutionary origin of floral organs of Erycina pusilla, an emerging orchid model system
Source: BMC Evol Biol. 2017 Mar 23;17:89. doi: 10.1186/s12862-017-0938-7 (PMC5364718; doi:10.1186/s12862-017-0938-7)
Supplement: Supplementary file 3 — Melting curve analysis of all primer pairs used in this study performed at the end of the PCR cycles to confirm the specificity of primer annealing. (PDF 9997 kb) [file 12862_2017_938_MOESM2_ESM.pdf]

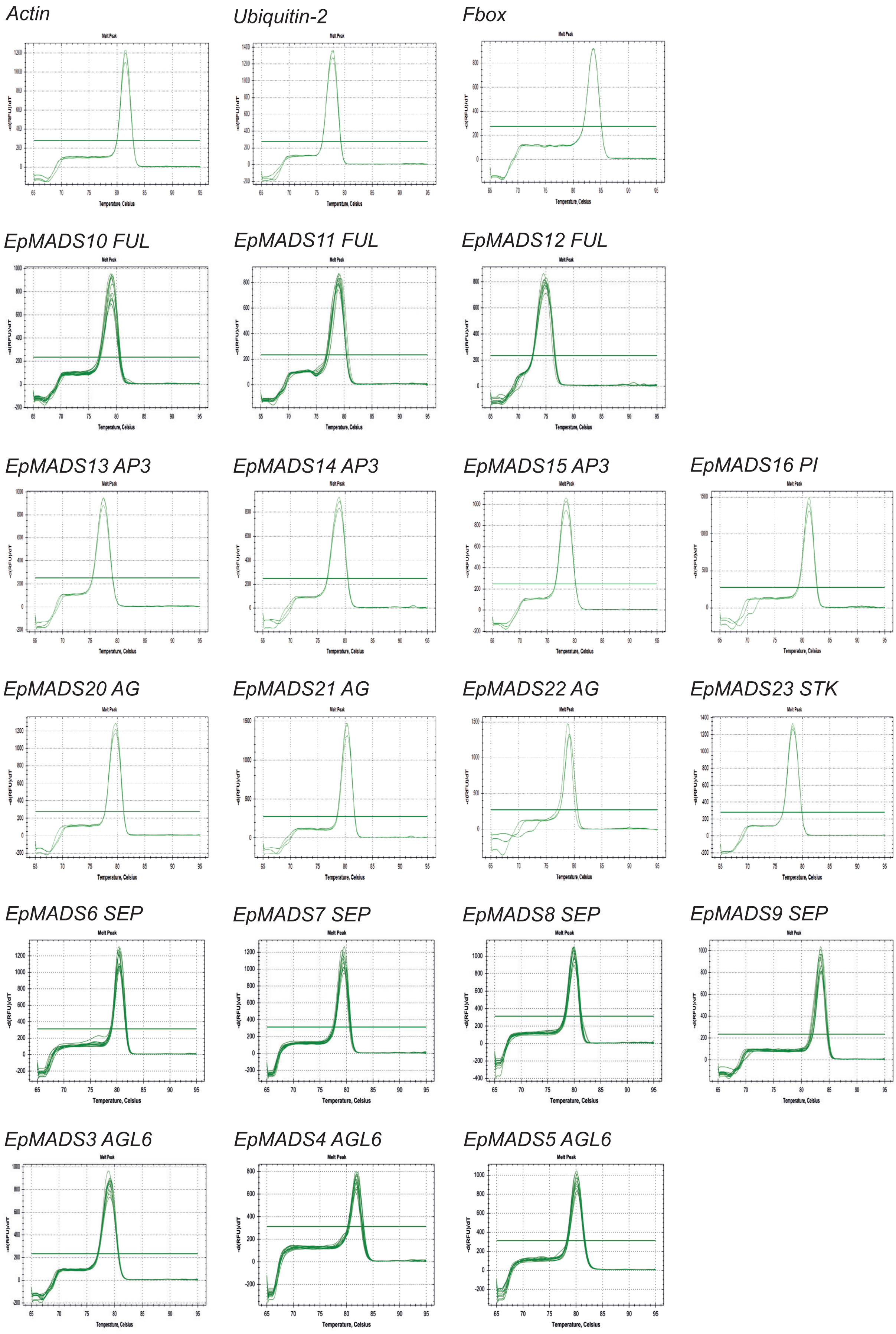

Actin
Ubiquitin-2
Fbox
EpMADS10 FUL
EpMADS11 FUL
EpMADS12 FUL
EpMADS13 AP3
EpMADS14 AP3
EpMADS15 AP3
EpMADS16 PI
EpMADS20 AG
EpMADS21 AG
EpMADS22 AG
EpMADS23 STK
EpMADS6 SEP
EpMADS7 SEP
EpMADS8 SEP
EpMADS9 SEP
EpMADS3 AGL6
EpMADS4 AGL6
EpMADS5 AGL6
Melt Peak
-d(RFU)/dT
Temperature, Celsius
