# Supplementary figures and images for: Exploring the evolutionary origin of floral organs of Erycina pusilla, an emerging orchid model system
Source: BMC Evol Biol. 2017 Mar 23;17:89. doi: 10.1186/s12862-017-0938-7 (PMC5364718; doi:10.1186/s12862-017-0938-7)

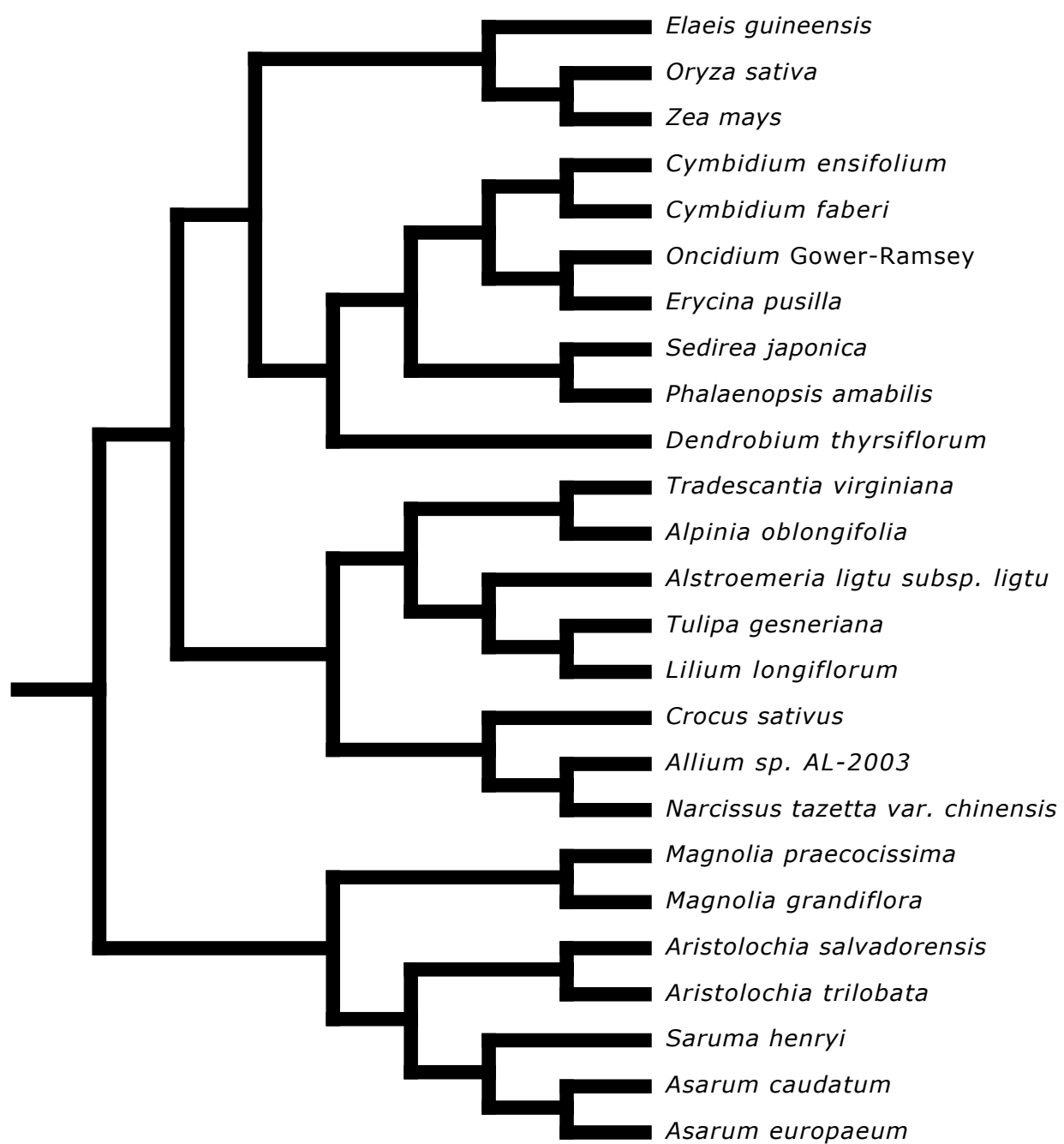

Supplement: Supplementary file 4 — Species phylogeny compiled based on Topik et al. [66], Biswal et al. [67], Takamiya et al. [68] and Chase et al. [69] for (a) FUL-, (b) AP3-, (c) PI- (d) AG- and STK-, (e) SEP- and (f) AGL6-like MADS-box gene lineage trees. (ZIP 584 kb) [file 12862_2017_938_MOESM3_ESM.zip › Dirks-Mulder Fig S4a.pdf]

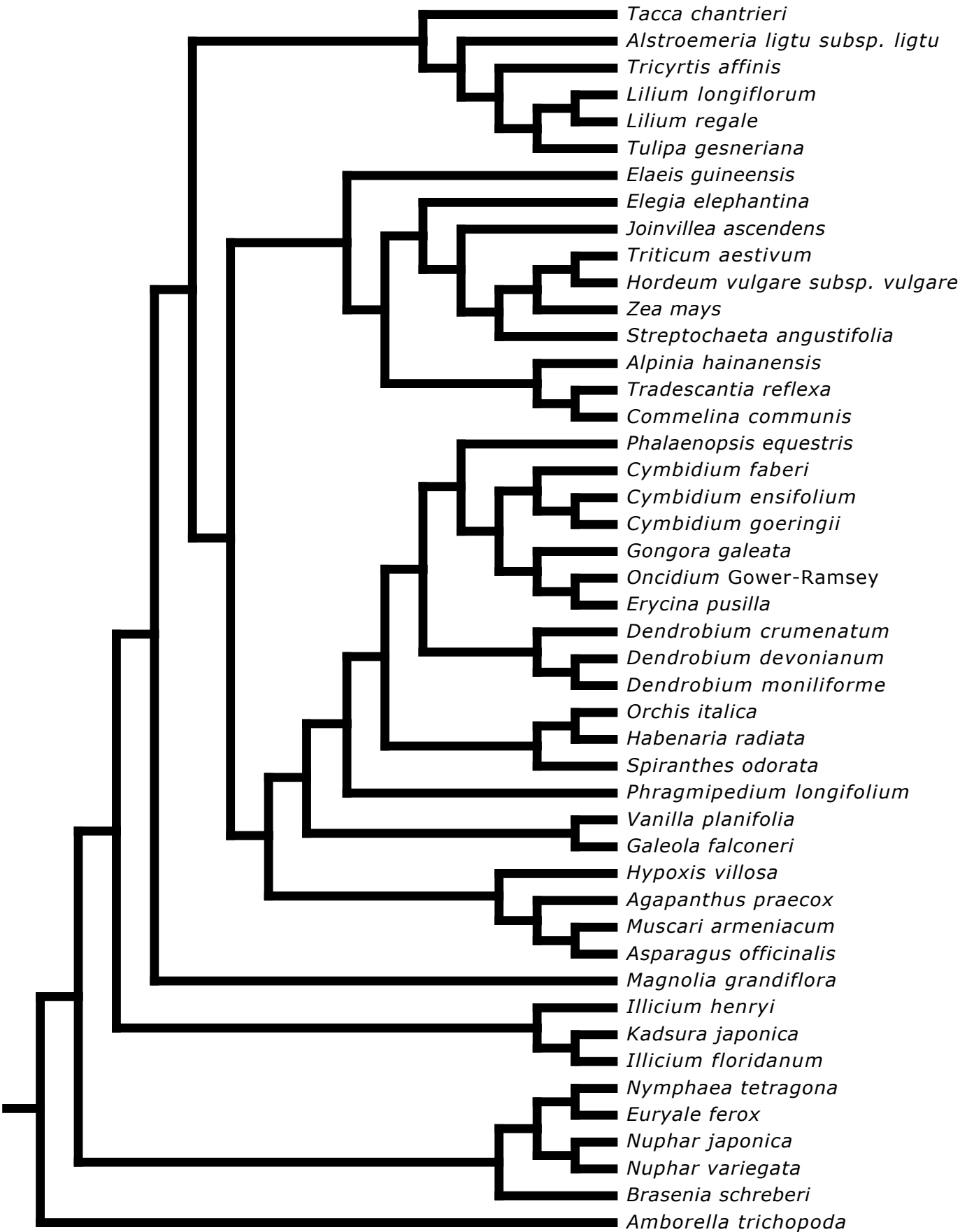

Supplement: Supplementary file 4 — Species phylogeny compiled based on Topik et al. [66], Biswal et al. [67], Takamiya et al. [68] and Chase et al. [69] for (a) FUL-, (b) AP3-, (c) PI- (d) AG- and STK-, (e) SEP- and (f) AGL6-like MADS-box gene lineage trees. (ZIP 584 kb) [file 12862_2017_938_MOESM3_ESM.zip › Dirks-Mulder Fig S4b.pdf]

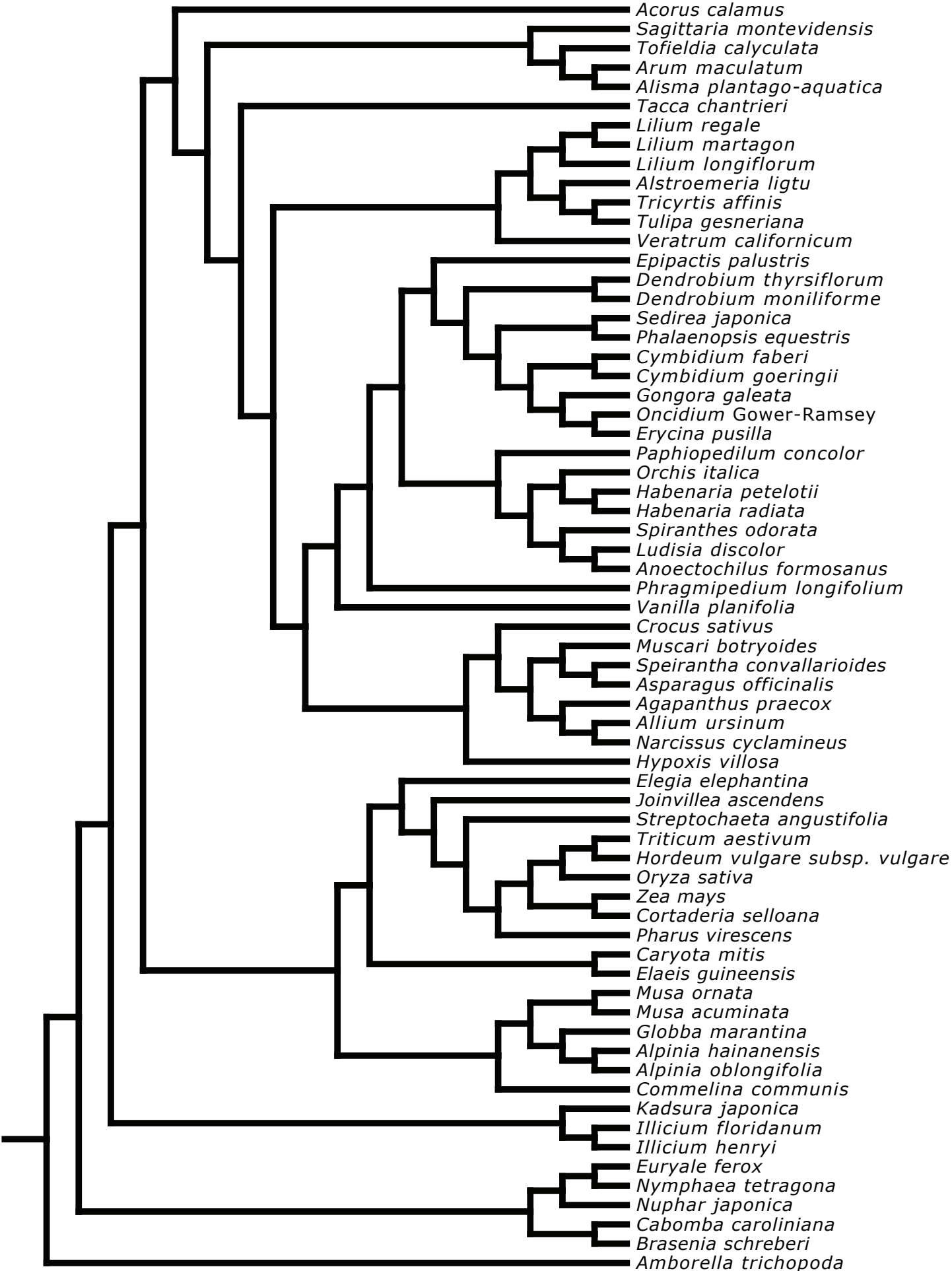

Supplement: Supplementary file 4 — Species phylogeny compiled based on Topik et al. [66], Biswal et al. [67], Takamiya et al. [68] and Chase et al. [69] for (a) FUL-, (b) AP3-, (c) PI- (d) AG- and STK-, (e) SEP- and (f) AGL6-like MADS-box gene lineage trees. (ZIP 584 kb) [file 12862_2017_938_MOESM3_ESM.zip › Dirks-Mulder Fig S4c.pdf]

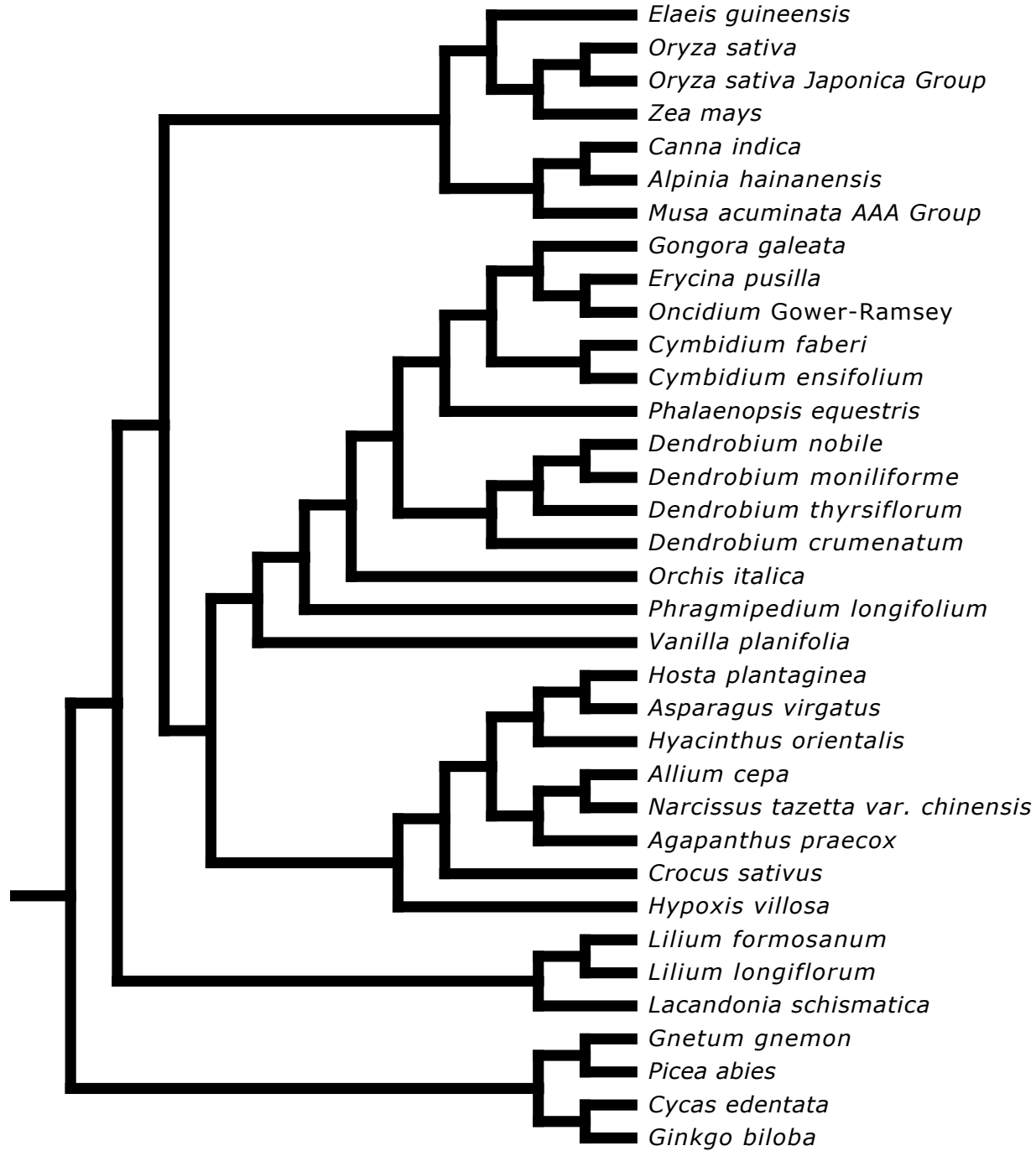

Supplement: Supplementary file 4 — Species phylogeny compiled based on Topik et al. [66], Biswal et al. [67], Takamiya et al. [68] and Chase et al. [69] for (a) FUL-, (b) AP3-, (c) PI- (d) AG- and STK-, (e) SEP- and (f) AGL6-like MADS-box gene lineage trees. (ZIP 584 kb) [file 12862_2017_938_MOESM3_ESM.zip › Dirks-Mulder Fig S4d.pdf]

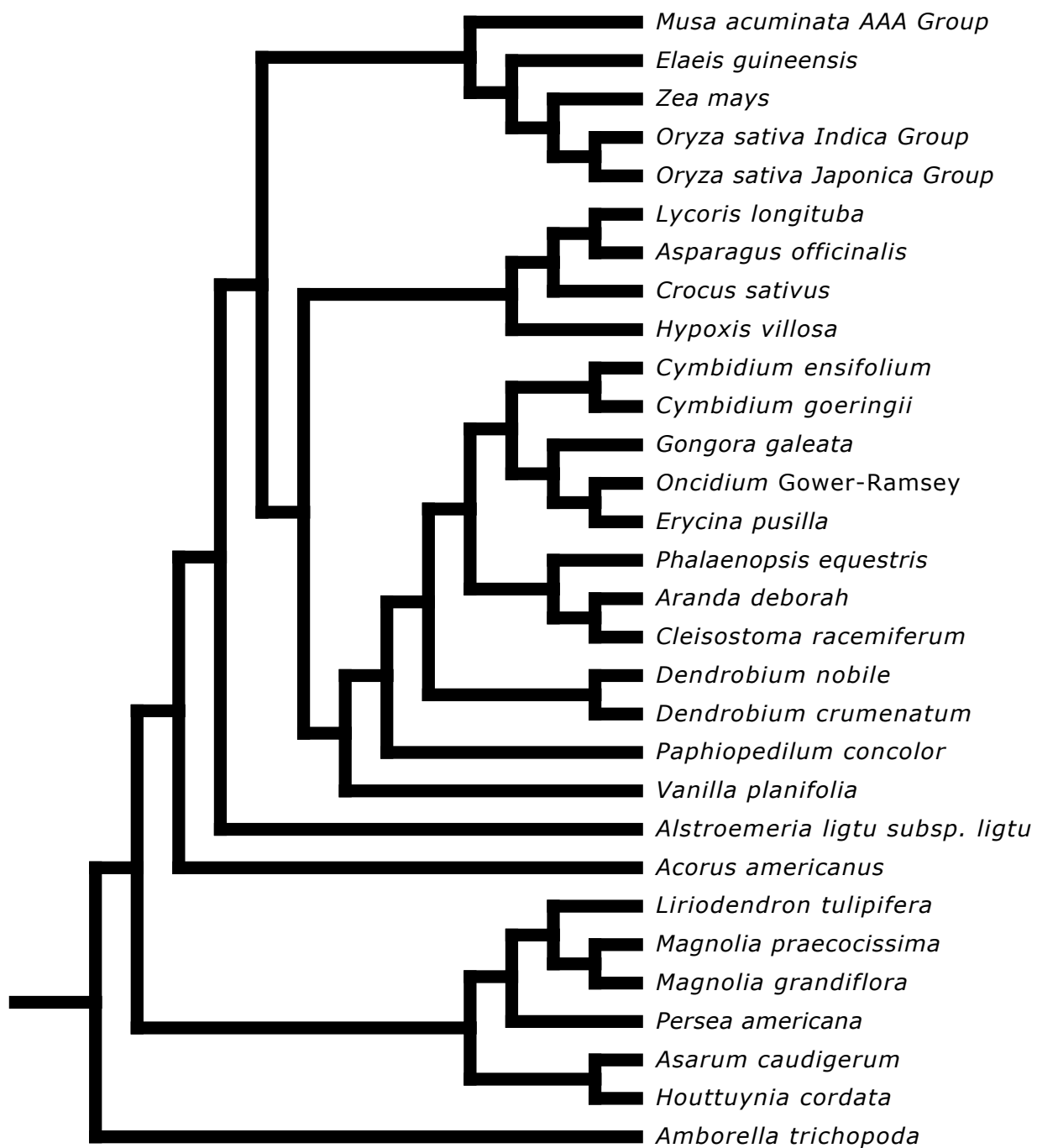

Supplement: Supplementary file 4 — Species phylogeny compiled based on Topik et al. [66], Biswal et al. [67], Takamiya et al. [68] and Chase et al. [69] for (a) FUL-, (b) AP3-, (c) PI- (d) AG- and STK-, (e) SEP- and (f) AGL6-like MADS-box gene lineage trees. (ZIP 584 kb) [file 12862_2017_938_MOESM3_ESM.zip › Dirks-Mulder Fig S4e.pdf]

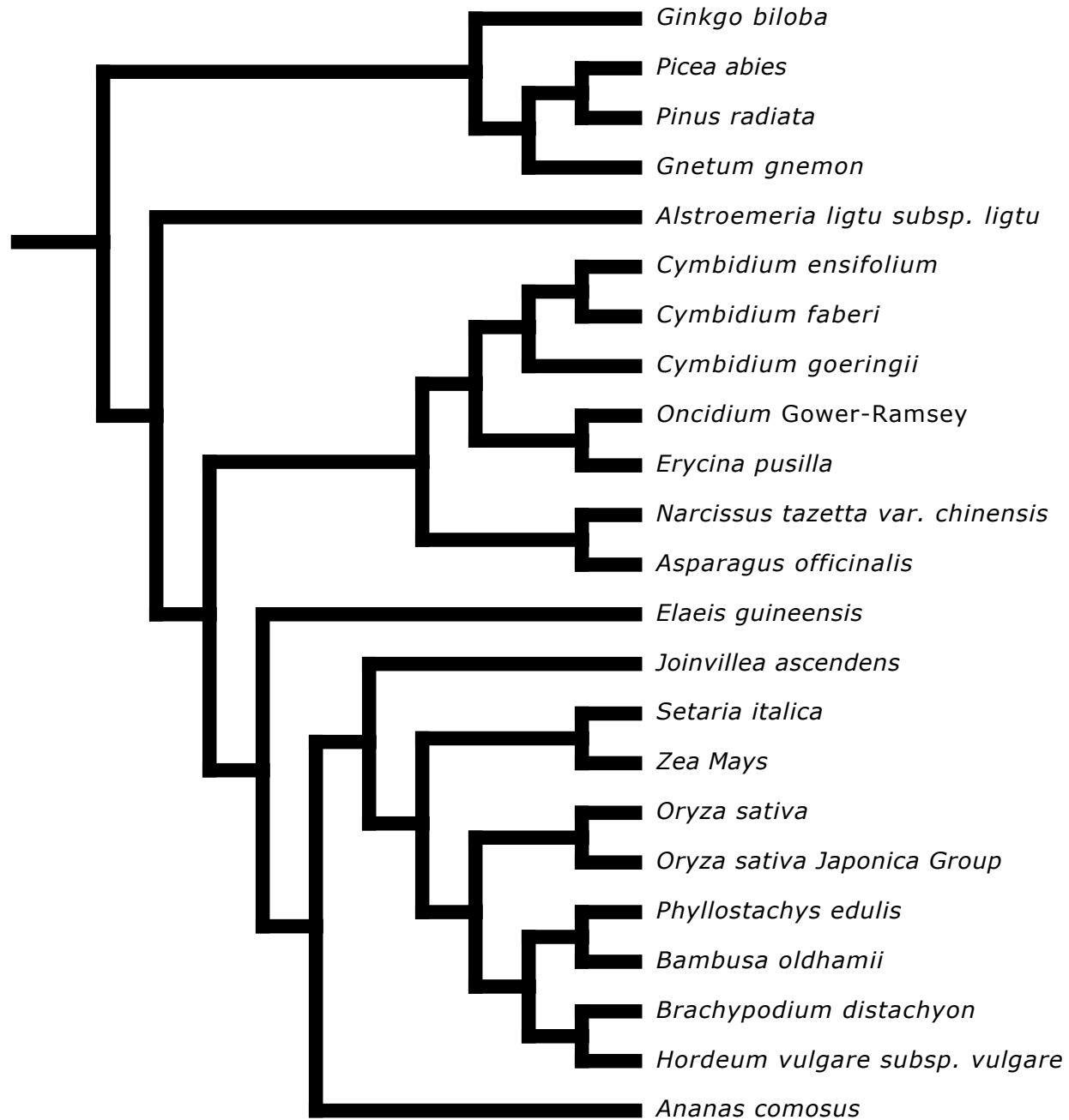

Supplement: Supplementary file 4 — Species phylogeny compiled based on Topik et al. [66], Biswal et al. [67], Takamiya et al. [68] and Chase et al. [69] for (a) FUL-, (b) AP3-, (c) PI- (d) AG- and STK-, (e) SEP- and (f) AGL6-like MADS-box gene lineage trees. (ZIP 584 kb) [file 12862_2017_938_MOESM3_ESM.zip › Dirks-Mulder Fig S4f.pdf]

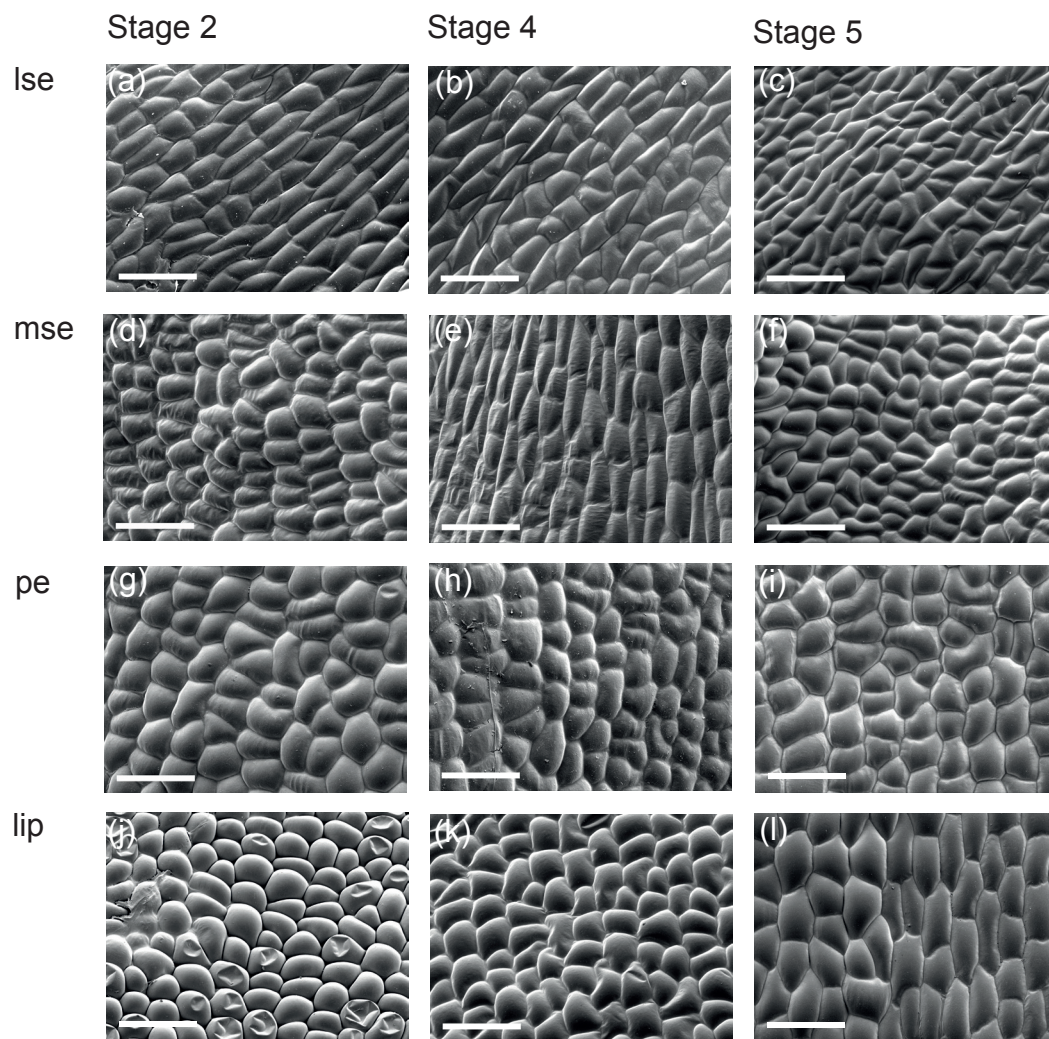

Supplement: Supplementary file 5 — Scanning electron micrographs of epidermal cells on the abaxial side of an E. pusilla flower. The three columns represent, from left to right, stage 2, 4 and 5 floral organs. Epidermal cells of (a–c) lateral sepal, (d–f) median sepal, (g–i) petal and (j–l) lip. Scale bar = 100 μm. Abbreviations: lse = lateral sepal; mse = median sepal; pe = petal. (PDF 19591 kb) [file 12862_2017_938_MOESM5_ESM.pdf]

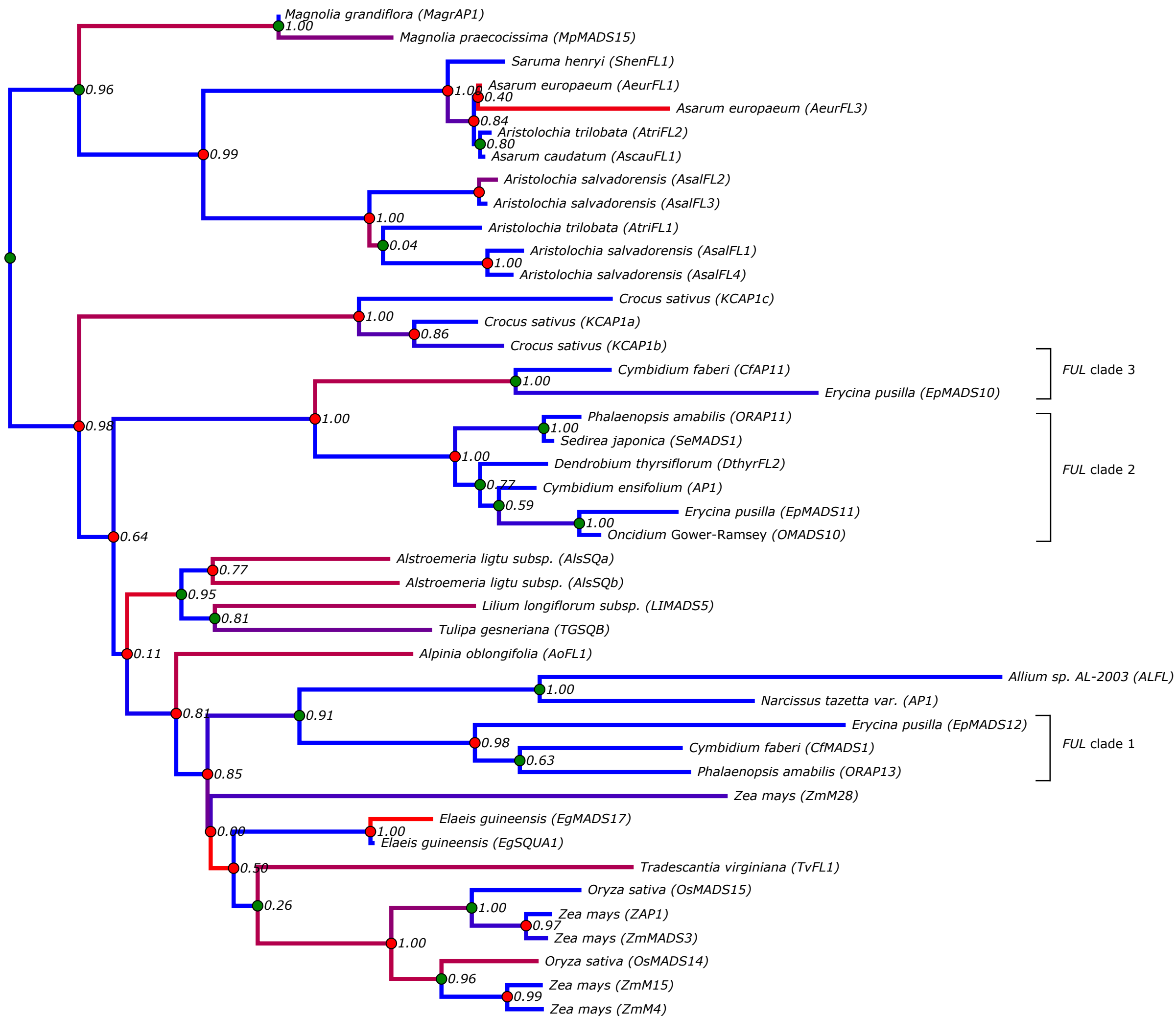

Supplement: Supplementary file 6 — MADS-box gene lineage trees. (a) FUL-, (b) AP3-, (c) PI-, (d) AG-, (e) STK-, (f) SEP- and (g) AGL6-like trees. Color codes: green node = speciation event; red node = duplication event. Branches are colored along a gradient between blue and red, in proportion to the value of omega (dN/dS) for the third (i.e. the highest) rate class in the BranchSiteREL analysis. Hence, blue and red branches may be interpreted as suggesting, respectively, stabilizing and diversifying selection. Purple branches implicate a moderate level of diversifying selection. (ZIP 1121 kb) [file 12862_2017_938_MOESM6_ESM.zip › Dirks-Mulder Fig S5a.pdf]

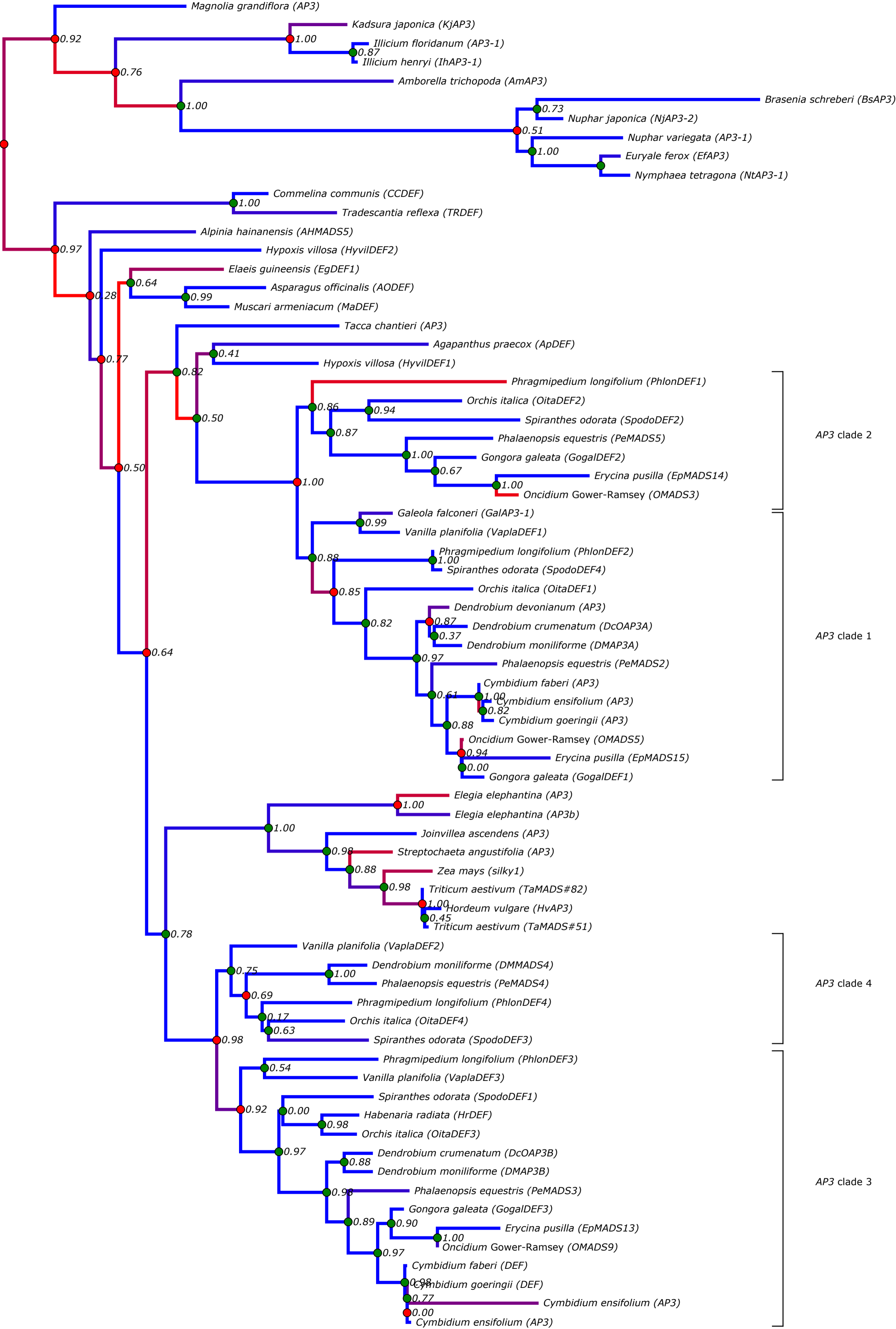

Supplement: Supplementary file 6 — MADS-box gene lineage trees. (a) FUL-, (b) AP3-, (c) PI-, (d) AG-, (e) STK-, (f) SEP- and (g) AGL6-like trees. Color codes: green node = speciation event; red node = duplication event. Branches are colored along a gradient between blue and red, in proportion to the value of omega (dN/dS) for the third (i.e. the highest) rate class in the BranchSiteREL analysis. Hence, blue and red branches may be interpreted as suggesting, respectively, stabilizing and diversifying selection. Purple branches implicate a moderate level of diversifying selection. (ZIP 1121 kb) [file 12862_2017_938_MOESM6_ESM.zip › Dirks-Mulder Fig S5b.pdf]

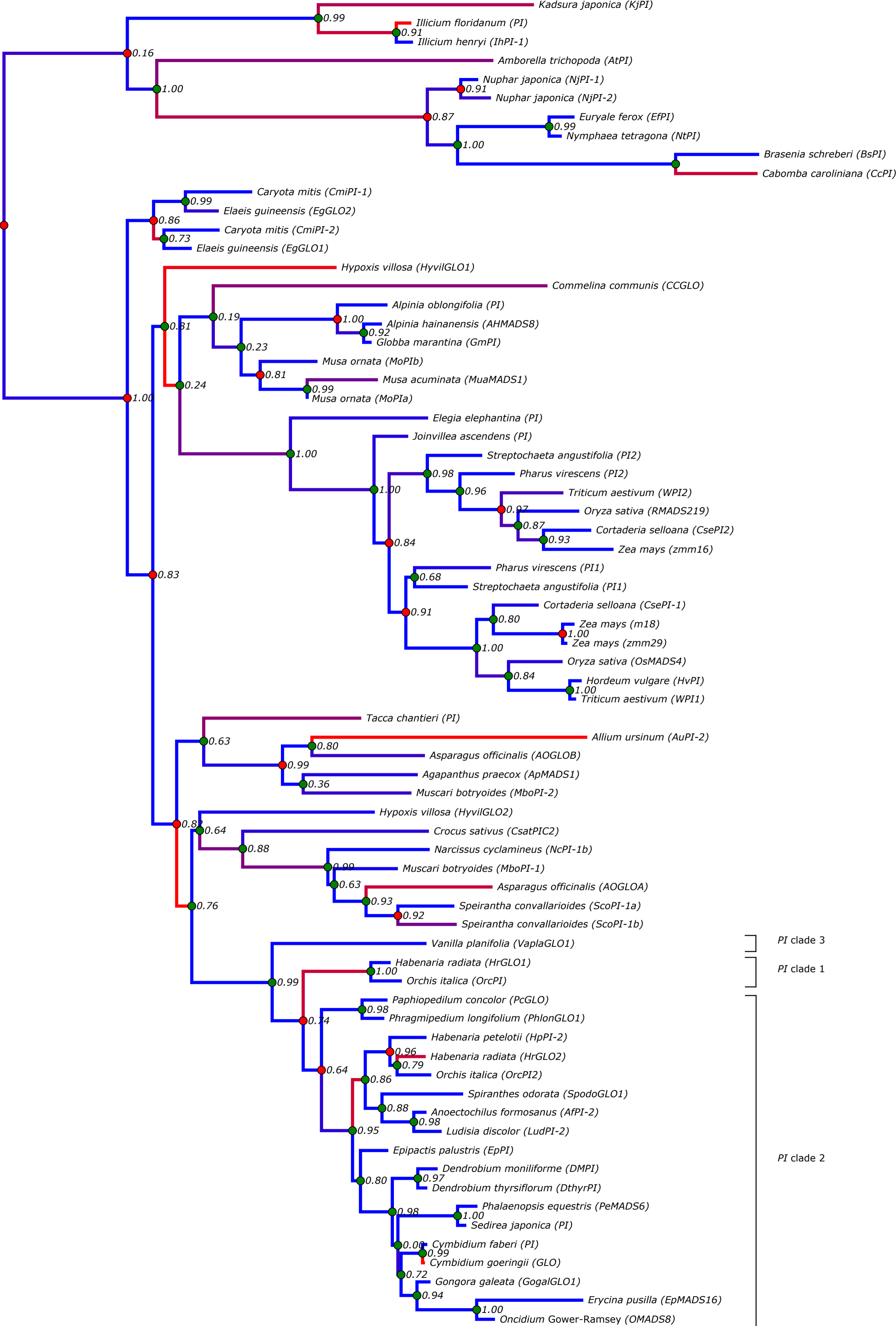

Supplement: Supplementary file 6 — MADS-box gene lineage trees. (a) FUL-, (b) AP3-, (c) PI-, (d) AG-, (e) STK-, (f) SEP- and (g) AGL6-like trees. Color codes: green node = speciation event; red node = duplication event. Branches are colored along a gradient between blue and red, in proportion to the value of omega (dN/dS) for the third (i.e. the highest) rate class in the BranchSiteREL analysis. Hence, blue and red branches may be interpreted as suggesting, respectively, stabilizing and diversifying selection. Purple branches implicate a moderate level of diversifying selection. (ZIP 1121 kb) [file 12862_2017_938_MOESM6_ESM.zip › Dirks-Mulder Fig S5c.pdf]

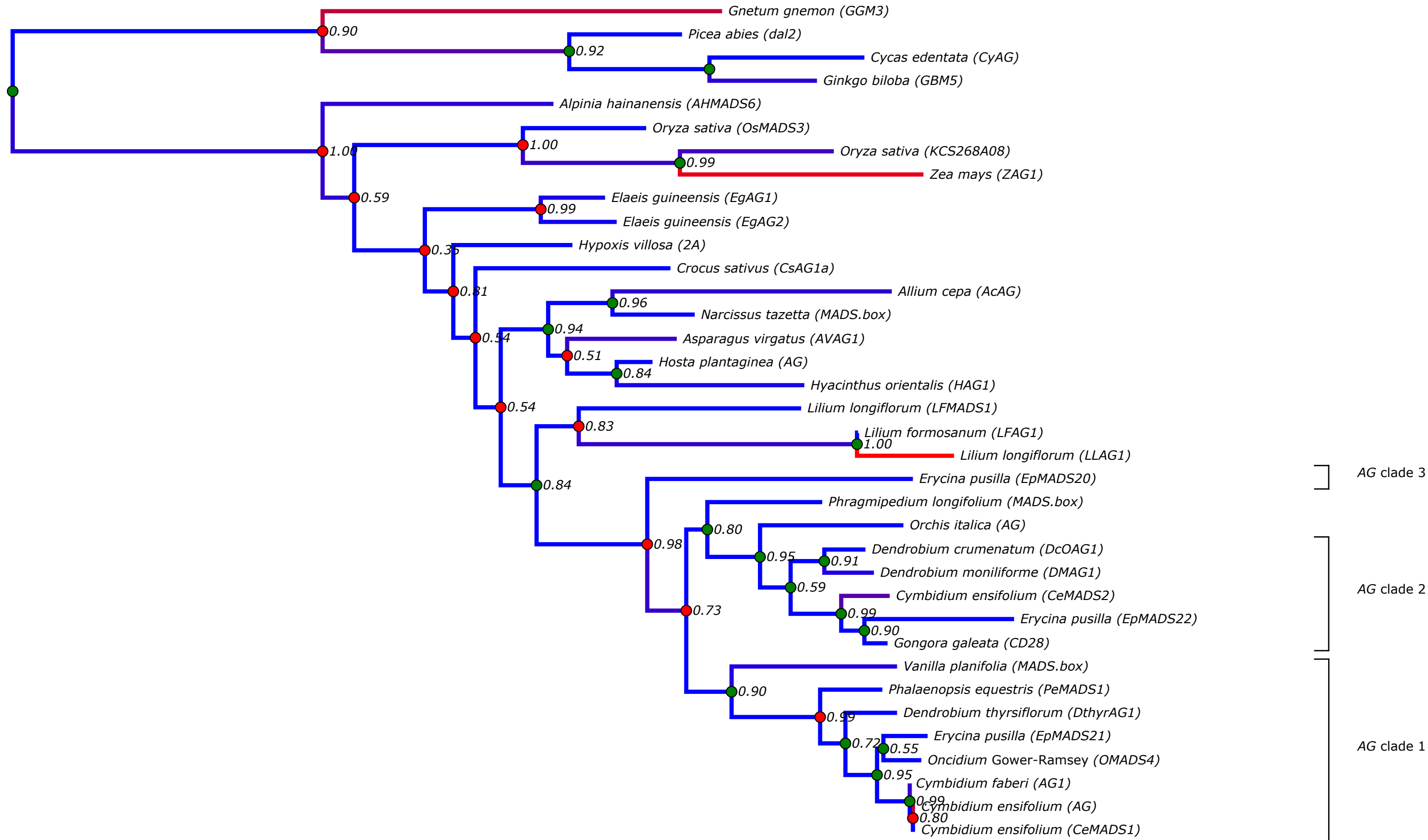

Supplement: Supplementary file 6 — MADS-box gene lineage trees. (a) FUL-, (b) AP3-, (c) PI-, (d) AG-, (e) STK-, (f) SEP- and (g) AGL6-like trees. Color codes: green node = speciation event; red node = duplication event. Branches are colored along a gradient between blue and red, in proportion to the value of omega (dN/dS) for the third (i.e. the highest) rate class in the BranchSiteREL analysis. Hence, blue and red branches may be interpreted as suggesting, respectively, stabilizing and diversifying selection. Purple branches implicate a moderate level of diversifying selection. (ZIP 1121 kb) [file 12862_2017_938_MOESM6_ESM.zip › Dirks-Mulder Fig S5d.pdf]

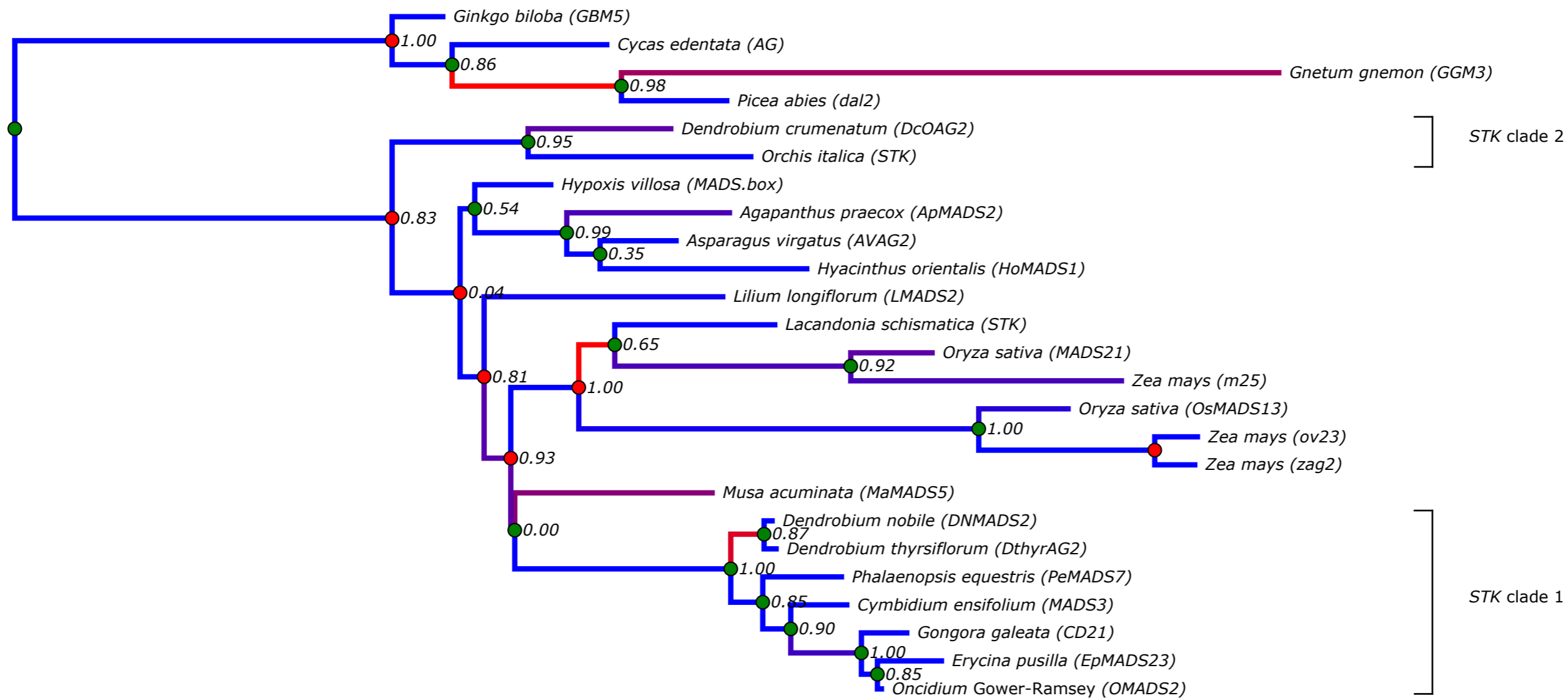

Supplement: Supplementary file 6 — MADS-box gene lineage trees. (a) FUL-, (b) AP3-, (c) PI-, (d) AG-, (e) STK-, (f) SEP- and (g) AGL6-like trees. Color codes: green node = speciation event; red node = duplication event. Branches are colored along a gradient between blue and red, in proportion to the value of omega (dN/dS) for the third (i.e. the highest) rate class in the BranchSiteREL analysis. Hence, blue and red branches may be interpreted as suggesting, respectively, stabilizing and diversifying selection. Purple branches implicate a moderate level of diversifying selection. (ZIP 1121 kb) [file 12862_2017_938_MOESM6_ESM.zip › Dirks-Mulder Fig S5e.pdf]

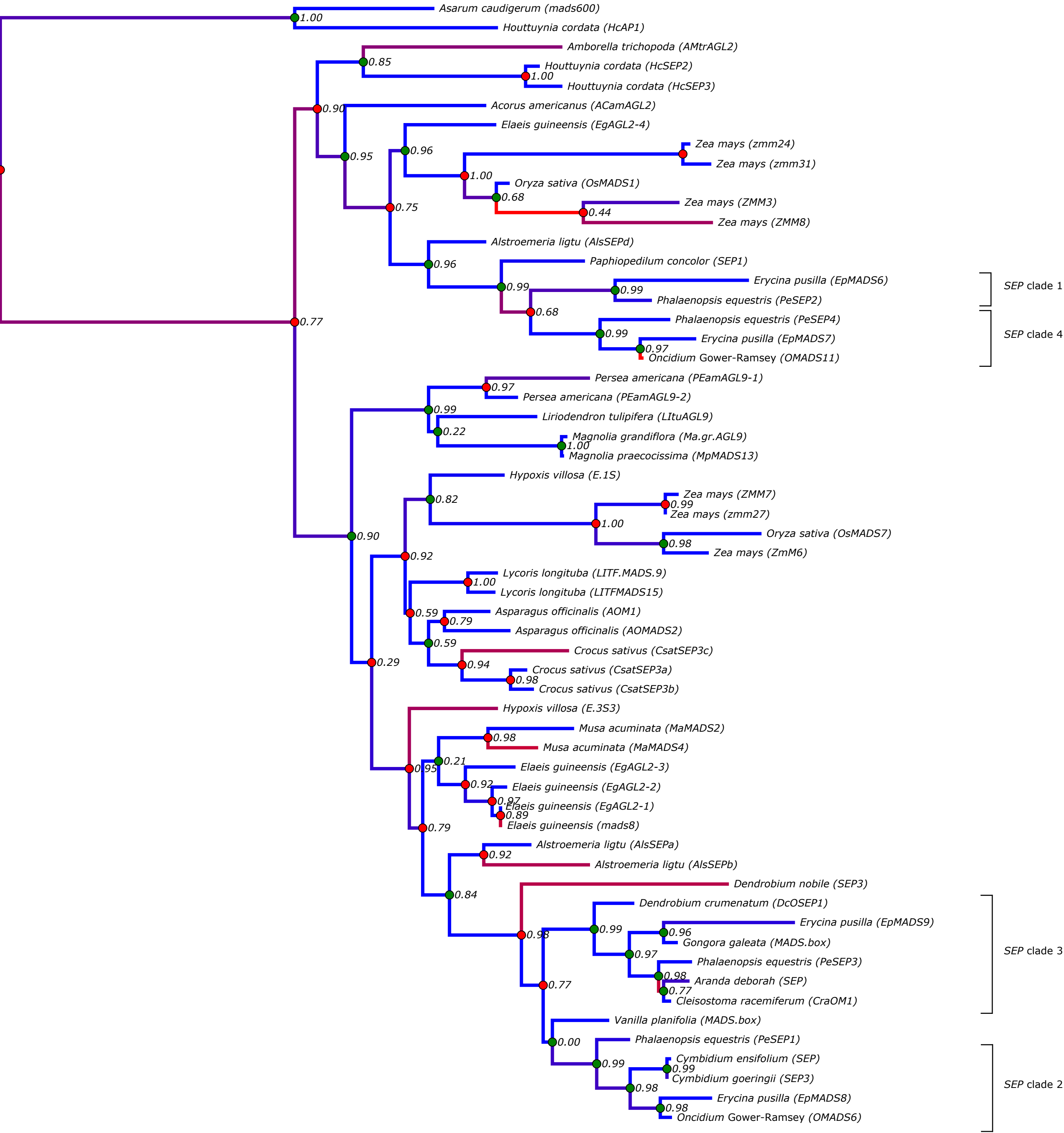

Supplement: Supplementary file 6 — MADS-box gene lineage trees. (a) FUL-, (b) AP3-, (c) PI-, (d) AG-, (e) STK-, (f) SEP- and (g) AGL6-like trees. Color codes: green node = speciation event; red node = duplication event. Branches are colored along a gradient between blue and red, in proportion to the value of omega (dN/dS) for the third (i.e. the highest) rate class in the BranchSiteREL analysis. Hence, blue and red branches may be interpreted as suggesting, respectively, stabilizing and diversifying selection. Purple branches implicate a moderate level of diversifying selection. (ZIP 1121 kb) [file 12862_2017_938_MOESM6_ESM.zip › Dirks-Mulder Fig S5f.pdf]

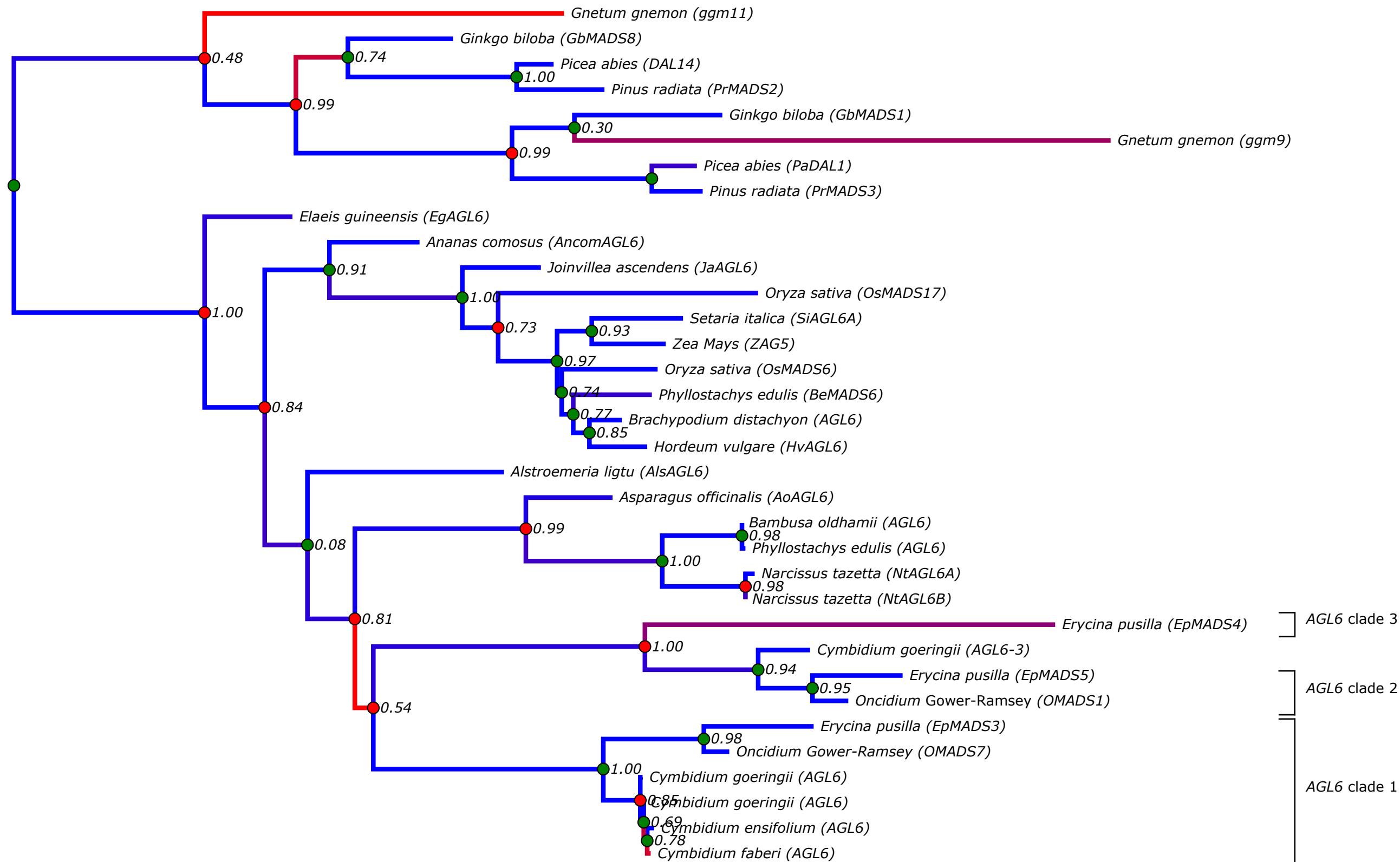

Supplement: Supplementary file 6 — MADS-box gene lineage trees. (a) FUL-, (b) AP3-, (c) PI-, (d) AG-, (e) STK-, (f) SEP- and (g) AGL6-like trees. Color codes: green node = speciation event; red node = duplication event. Branches are colored along a gradient between blue and red, in proportion to the value of omega (dN/dS) for the third (i.e. the highest) rate class in the BranchSiteREL analysis. Hence, blue and red branches may be interpreted as suggesting, respectively, stabilizing and diversifying selection. Purple branches implicate a moderate level of diversifying selection. (ZIP 1121 kb) [file 12862_2017_938_MOESM6_ESM.zip › Dirks-Mulder Fig S5g.pdf]

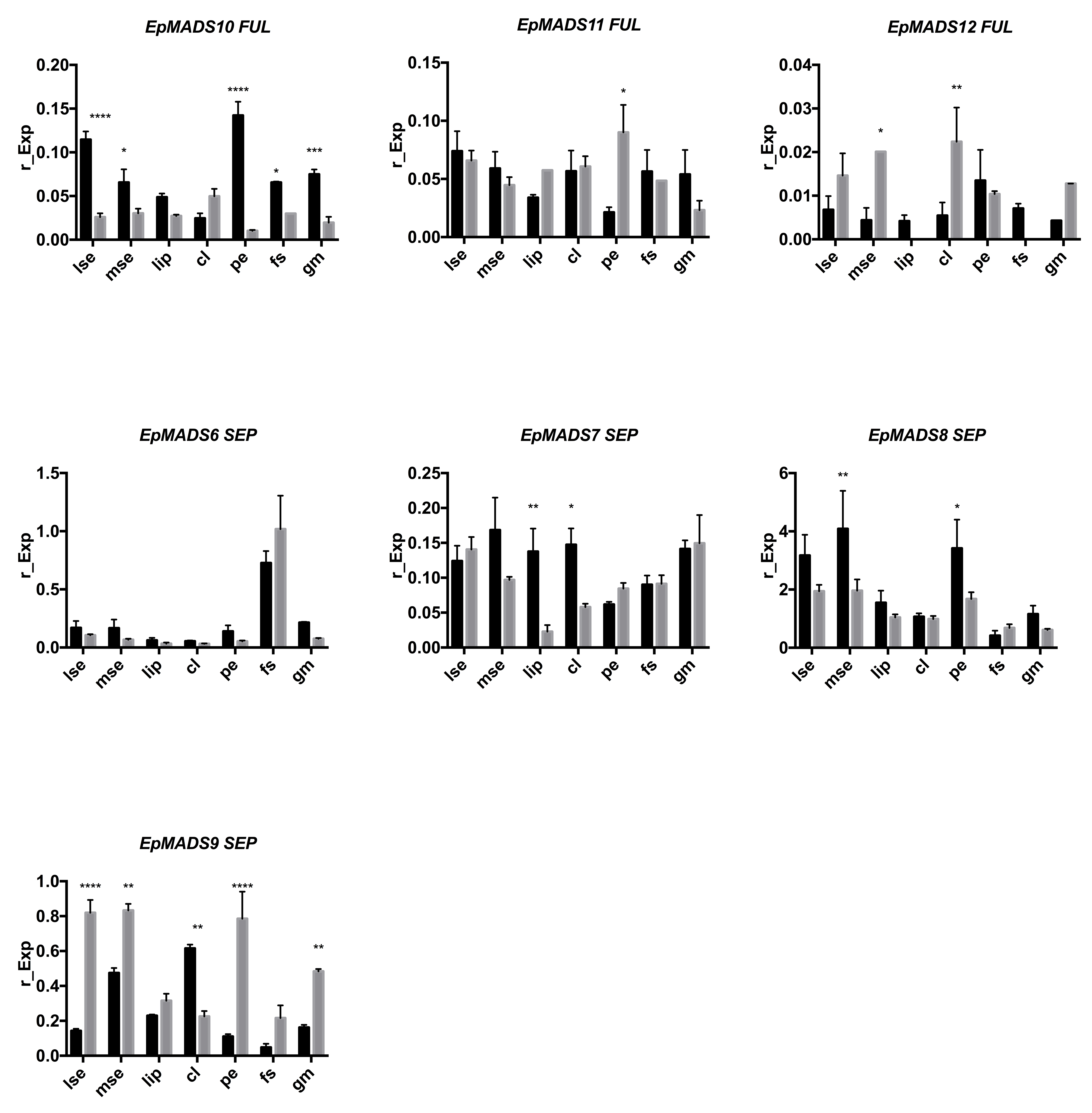

Supplement: Supplementary file 7 — Floral organ specific expression levels of FUL EpMADS10, EpMADS11 and EpMADS12 and SEP EpMADS6, EpMADS7, EpMADS8 and EpMADS9. RNA was extracted from seven different floral organs during two stages of development of E. pusilla and used for cDNA synthesis. Expression of the MADS-box genes was normalized to the geometric mean of three reference genes Actin, UBI2 and Fbox. Each column shows the relative expression of 20 floral organs in two cDNA pools (10 floral organs per isolation), both tested in triplicate. Abbreviations: lse = lateral sepal; mse = median sepal; cl = callus; pe = petal; fs = fertile stamen; gm = gynostemium. Dark grey = floral stage 2 and light grey = floral stage 4. Y-axis: relative gene expression. The error bars represent the Standard Error of Mean. P-value style: GP: >0.05 (ns), <0.05 (*), <0.01 (**), <0.001 (***), <0.0001 (****). (TIFF 3032 kb) [file 12862_2017_938_MOESM7_ESM.tiff]
